# Quantitative multiplexing with nano-self-assemblies in SERS

**DOI:** 10.1038/srep06785

**Published:** 2014-10-30

**Authors:** Setu Kasera, Lars O. Herrmann, Jesús del Barrio, Jeremy J. Baumberg, Oren A. Scherman

**Affiliations:** 1 Melville Laboratory for Polymer Synthesis, Department of Chemistry; 2 Nanophotonics Centre, Cavendish Laboratory, University of Cambridge, UK

## Abstract

Multiplexed or simultaneous detection of multiple analytes is a valuable tool in many analytical applications. However, complications caused by the presence of interfering compounds in a sample form a major drawback in existing molecular sensor technologies, particularly in multi-analyte systems. Although separating analytes through extraction or chromatography can partially address the problem of interferents, there remains a need for developing direct observational tools capable of multiplexing that can be applied *in situ*. Surface-enhanced Raman Spectroscopy (SERS) is an optical molecular finger-printing technique that has the ability to resolve analytes from within mixtures. SERS has attracted much attention for its potential in multiplexed sensing but it has been limited in its quantitative abilities. Here, we report a facile supramolecular SERS-based method for quantitative multiplex analysis of small organic molecules in aqueous environments such as human urine.

SERS is a highly sensitive surface technique that enhances inherently weak Raman signals from molecules that are localised in regions of intense optical fields trapped between adjacent plasmonic surfaces, called ‘hot-spots'^1^. Noble metal nanoparticles are favoured for small molecule analysis as they allow *in situ* detection within liquid media, compatible with microfluidic devices^2^. Furthermore, their low cost, commercial availability and ease of synthesis without the need for sophisticated instruments renders them practical for widespread use.

In the last few years, the realisation that SERS is an ideal technique for analysis of multiple analytes simultaneously, i.e. multiplexing, has drawn considerable interest. Multiplexing is desirable to eliminate the steps that are often required to isolate the analyte of interest from a complex mixture of compounds^3^. Despite its advantages, the quantification of SERS, especially for multiplexing, has been challenging as a result of irreproducible SERS intensities^4^,^5^. While *semi-quantitative* methods have been widely reported in the literature, there are no examples of supramolecular SERS-based quantitative multiplexed methods^5^,^6^,^7^,^8^,^9^.

Several approaches have focused on obtaining highly-controlled SERS signals, for instance through using DNA-oligomers^10^,^11^, alignment of nanoparticle arrays at multi-phase interfaces^12^ and others^4^. One such strategy, applicable in aqueous media, utilises rigid spacer macrocyclic host molecules, cucurbit[*n*]urils^13^ (CB[*n*]), to create precisely spaced sub-nanometre gaps in between adjacent nanoparticles^14^,^15^. The resultant reproducible hot-spot regions generate quantitative SERS signals^16^. In addition, analytes of interest can be localised near the surface of the gold nanoparticles through their affinity to the CB[*n*] cavity^17^. Therefore, CB[*n*] provides an ideal supramolecular approach for the generation of quantitative SERS signals.

CB[7] can accommodate an aromatic compound inside its cavity to form 1:1 guest·CB[7] complexes in aqueous solutions (Figure 1a). Host-guest complex formation with CB[7] is mainly driven by the release of high-energy water from inside the hydrophobic cavity of CB[7]^18^. Further electrostatic interactions between cationic guest molecules and the carbonyl portals of CB[7] lead to additional stabilisation of such complexes. CB[7] is selective towards a class of molecules instead of being specific for single target analytes only. Such a generic receptor is ideal for the development of a multiplexed chemosensor, where several structurally-similar compounds can be resolved spectroscopically, as in SERS.

A number of neurotransmitters act as disease biomarkers and are common analytes of interest in medical diagnostics, including microdialysates and urine samples^19^,^20^. In order to demonstrate the potential of this CB[7]-mediated SERS system in multiplexed chemical analysis, three such monoamine neurotransmitters were chosen for this study: dopamine (DA), epinephrine (EPI) and serotonin (5HT). Electrochemical methods and mass spectroscopy are the most conventional choices for the detection of these neurotransmitters^21^, while optical methods include fluorescent labelling using specifically designed molecular tags^22^. However, such methods are often impractical for real sample analyses as they are limited by their need for additional separation strategies and their lack of ability to identify (or quantify) multiple analytes simultaneously for the direct detection of these neurotransmitters in complex environments^20^. Enzyme-linked immunosorbent assay systems have also recently been reported for the detection of dopamine^23^ but such current state-of-the-art techniques are still limited by drawbacks such as low applicability in multi-analyte systems, low availability of antibody reagents, long time-scales for assay development (multiple days to weeks), sensitivity to storage conditions and reduced specificity in the presence of structural analogues of the target analytes^6^. Herein, we show an exemplary study with the aforementioned three biogenic amines to highlight the applicability of the facile CB[*n*]-based SERS sensing for multiplexing.

## Results

### Binding of CB[7] with analytes

**Figure 1:**
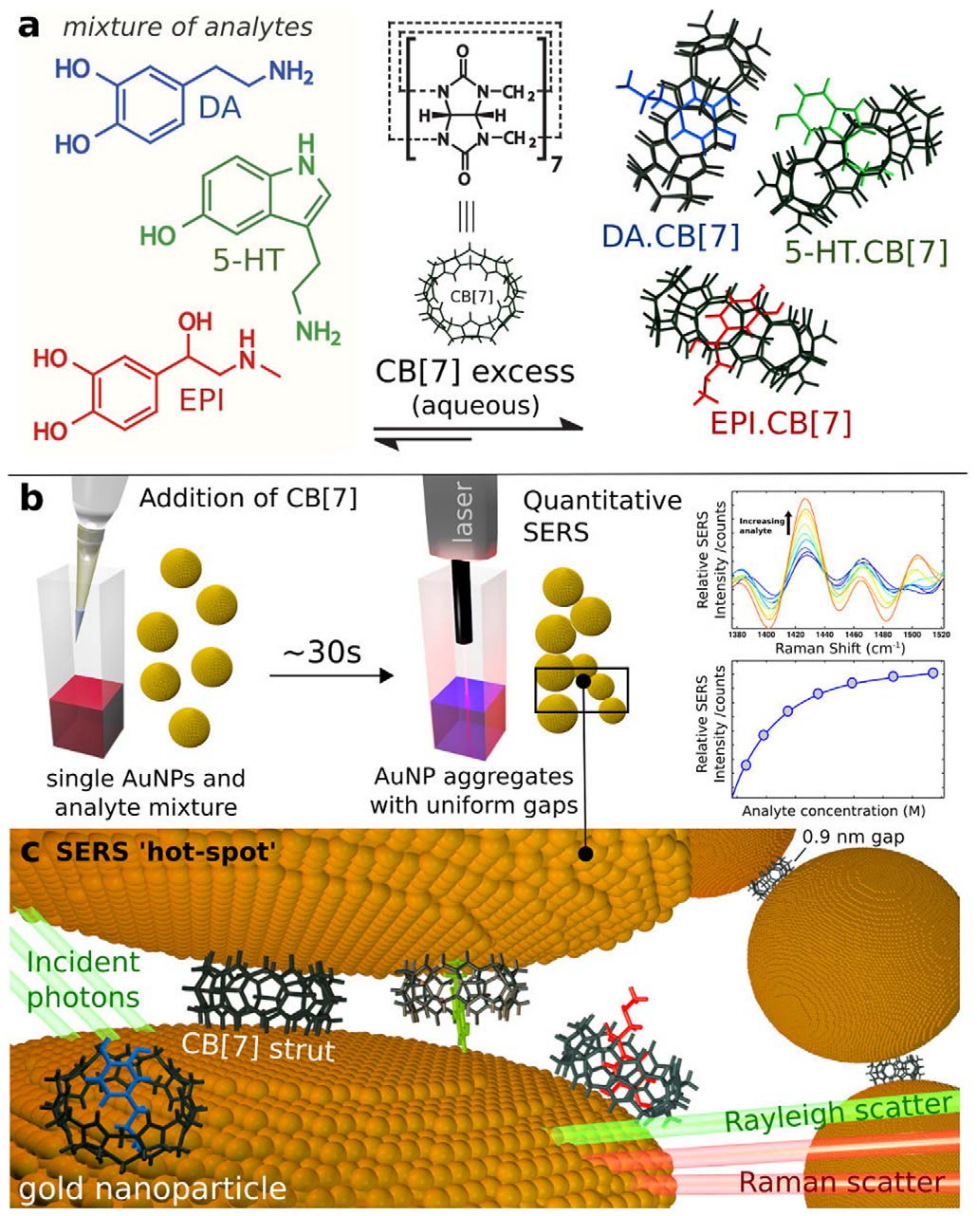
Conceptual schematic of the SERS-based multiplexed sensor. (a), Host-guest chemistry: epinephrine (EPI), dopamine (DA) and serotonin (5HT) form 1:1 inclusion complexes with macrocyclic host, cucurbit[7]uril (CB[7]). (b), Addition of CB[7] to gold colloids immediately bridges adjacent nanoparticles to create uniform gap distances between them, yielding precise hot-spots, which allow for instant quantitative SERS measurement. Analytes already present in the colloidal solution get localised in the hot-spot through their affinity for the CB[7] cavity (c), Schematic showing the localisation of guest molecules near the gold surface in the hot-spot through their encapsulation inside the CB[7] cavity.

DA and EPI are structurally similar molecules with catecholamine frameworks, whereas 5HT contains an indole moiety (Figure 1a). The three neurotransmitters show relatively similar binding affinities for CB[7] (10^4^ to 10^5^ M^−1^), as determined by NMR studies (Supplementary Figures S1–S4) and isothermal titration calorimetry (Supplementary Figure S5). All three monoamines bind to CB[7] with a 1:1 stoichiometry. In water (at pH 7), the amine groups on the neurotransmitters are protonated and contribute to the binding with CB[7] through stabilizing electrostatic interactions with the carbonyl portals (Supplementary Note S.1.2).

**Figure 2:**
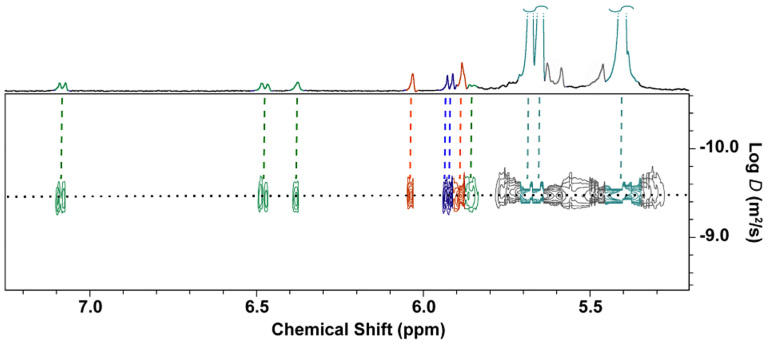
DOSY ^1^H NMR spectra of mixtures of neurotransmitters (dopamine, epinephrine and serotonin) and cucurbit[7]uril in a 1:1:1:3 ratio. All the neurotransmitters are bound to CB[7] in a 1:1 binding ratio when CB[7] is present in excess. The three host-guest complexes (EPI·CB[7], DA·CB[7] and 5HT·CB[7]) diffuse with similar diffusion coefficient values.

When EPI, DA and 5HT are present together in an aqueous solution containing excess CB[7], the binding behaviour of CB[7] towards the three individual analytes is not affected, as evidenced by the Diffusion Ordered Spectroscopy ^1^H NMR of their complexed mixture (Figure 2). The presence of excess CB[7] is key in this case. In the absence of sufficient concentration of CB[7] for all three neurotransmitters, the guests compete for the host with similar affinities, which results in unbound neurotransmitters in the solution (Supplementary Figure S6).

### Aggregation of nanoparticles

DA, EPI and 5HT are unable to aggregate gold nanoparticles (AuNPs) at low concentrations (≤5 × 10^−5^ M) and therefore, cannot be analysed by SERS directly in the absence of an aggregating agent. However, with subsequent addition of CB[7], AuNP cluster formation is induced with uniform gap distances between adjacent nanoparticles^14^ (Figure 1b). This allows for observation of SERS signals from the analytes well below 5 × 10^−5^ M (limit of detection < 10^−9^ M), which get trapped inside the CB[7] cavity (Figure 1c, Supplementary Figure S7). The kinetics of CB[*n*]-induced AuNP aggregation has been shown to be fully reproducible using fixed concentrations of CB[*n*] and aqueous nanoparticles^14^,^16^. The clusters are formed within a few seconds after addition of CB[7], immediately enabling SERS data acquisition. In this particular study, SERS spectra were acquired after approximately 30 seconds of addition of CB[7] and acquisition was completed within the first minute of aggregation. However, it is noteworthy that SERS intensities remain stable over time and only show a slight decrease over 30 minutes (Supplementary Figures S8–S9). Therefore, measurements do not have to be taken at precise or exact time points after addition of CB[7] and spectra can be recorded within several minutes after the aggregation process has been initiated.

### SERS data analyses

**Figure 3:**
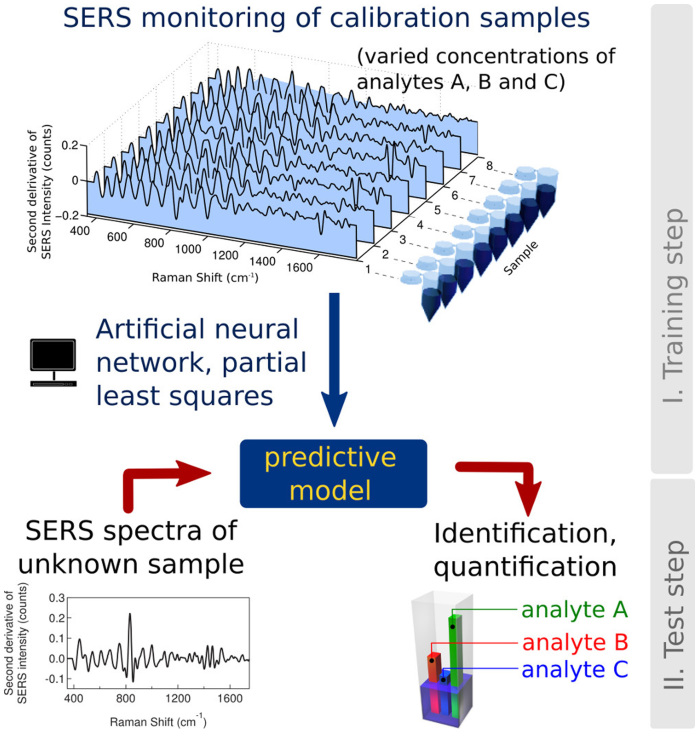
Schematic representing the experimental and analytical steps. At first, SERS data is collected from a series of calibration samples with different known concentrations of the analytes. This data is then used to ‘train' the data mining methods to build a predictive model. In the next step, SERS spectra of an unknown sample is then collected and analysed using the predictive model to obtain a result.

**Figure 4:**
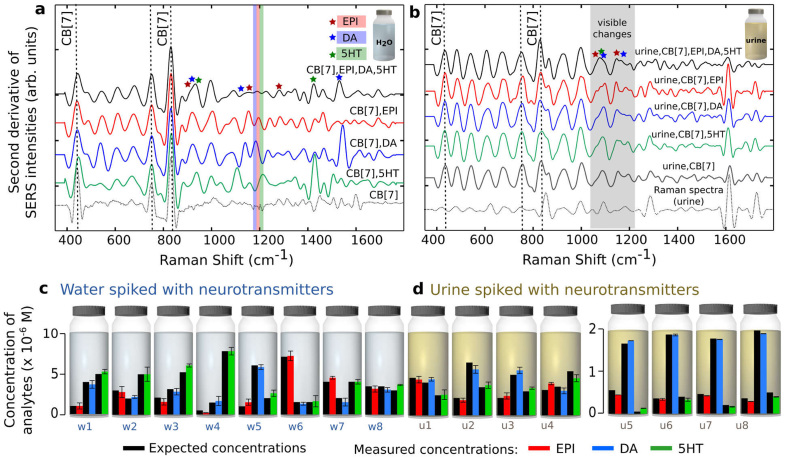
SERS analysis using the supramolecular CB[7]-gold nanoparticle sensor. (a), SERS spectra in H_2_O showing prominent visible CB[7] and neurotransmitter signals collected from the respective mixtures with a CB[7] to guest ratio of 5 (CB[7] concentration 1 × 10^−5^ M). (b), SERS spectra in urine highlighting visible CB[7] signals and noticeable changes in indicated spectral regions for urine samples spiked with neurotransmitters. It is noteable that visual spectral analyses becomes increasingly challenging with increasing number of analytes. Representative predicted concentrations of EPI, DA and 5HT in (c), water and (d), urine media (the last four bottles represent clinically relevant concentrations). Error bars represent absolute standard deviation calculated from 3 sample measurements. (Note: Second derivative of SERS spectra were taken to remove linear part of the SERS background without fitting; the spectra was normalised using the main CB[7] vibration at 830 cm^−1^ and have been stacked for clarity.)

While each analyte can be individually detected and quantified by SERS when analysed separately (Supplementary Figures S10–S11), the presence of multiple analytes in a mixture obscures the SERS spectra and visual inspection of such data becomes difficult (Supplementary Figures S12–S13). Numerical chemometric methods simplify the spectral deconvolution process by using relevant calibration or ‘training' data^24^. The principle of the analysis is analogous to the widely used analytical approach, where a calibration curve of known magnitude is prepared as a reference for the determination of unknown values in subsequent test measurements (Figure 3). It is particularly suited to extract quantitative information from spectra with multiple peaks where visual inspection is difficult (Figure 4a,b). At first a set of SERS data comprising triplicates of thirty different aqueous mixtures was collected for training the predictive numerical models, where the concentrations of DA, EPI and 5HT were varied, while the concentration of CB[7] was held constant. A broad range of concentrations were chosen, between 0.5 × 10^−6^ M and 10 × 10^−6^ M, to include expected clinical uretic concentrations of the neurotransmitters. This dataset formed the equivalent ‘calibration curve' in this study. A second set of data, or the ‘test set', was then collected comprising aqueous mixtures of neurotransmitters at randomly selected concentrations.

Firstly, the presence or absence of an analyte from the test mixtures was determined by Artificial Neural Networks (ANNs)^25^,^26^. ANNs are inspired by and imitate natural neural networks. They are widely used in pattern recognition and classification of specimens into known classes^26^. ANNs consist of interconnected neurons, or ‘nodes', arranged into input, intermediate (hidden) and output layers. There may be one or more intermediate layers, the number of which is determined based on a residual analysis during the training process. The connections between nodes are assigned random weights at first. During the training or ‘learning', these weights are iteratively adjusted when the algorithm is presented with an input pattern and a corresponding output pattern by comparing their differences. This process is repeated until the computed output matches the desired output. The resulting optimised or ‘trained' network is then used to determine the unknown output parameter using a set of measured input data.

For this study, a simple ‘three-layer feed-forward network', consisting of one hidden layer was used. Using the calibration data collected earlier, the algorithm was trained such that values of ‘0' indicate the absence and values of ‘1' the presence of a component. Such qualitative tests are common in screening illicit drugs, particularly in urine, using immunoassays. The results showed that it was possible to correctly detect the presence or absence of an analyte in a mixture, the neurotransmitters in this case, by application of ANNs on the spectral data of the mixtures collected with CB[7] (Supplementary Figure S14). Out of a total of 24 predictions (for 3 analytes in 8 samples), 22 predictions were correct, while 2 results were false positives (Supplementary Note S.4.1). This represents a 92% prediction accuracy, which is comparable to immunoassays. It must be noted that all positive results in drug screening remain presumptive until confirmed by a secondary method such as gas chromatography^27^,^28^. Therefore, the obtained results highlight the potential of this SERS-based method in preliminary qualitative screening of analytes.

Next, the potential of this method for measuring absolute concentrations of individual analytes was examined. After an initial comparison with other multivariate methods, namely Principle Component Regression (PCR), the well-established Partial Least Squares Regression (PLSR)^29^ method was chosen for further studies on account of better performance^30^. The same training and test datasets were used for the analyses as before. In brief, PLSR is a well-established multivariate regression method that can be used to extract sample concentrations from optical spectra^31^,^32^. It builds a predictive model that is based on the underlying factors that are responsible for the majority of the variation in the experimentally observed spectra. At the same time, it ensures that each of those factors is directly related to the analyte concentrations. Factors that only explain small variations in the spectrum such as noise are excluded from the model^29^. This reduction to relevant factors makes the predictive abilities of a PLSR model more robust compared to a simple linear regression model.

The PLSR model was trained using the calibration data collected earlier and validated with the ‘test set'. The average error in the measured absolute concentrations of the analytes in the test samples was within ±6 × 10^−7^ M of the expected concentrations in the test range between 0.5 × 10^−6^ M and 10 × 10^−6^ M. (Figure 4c, see Supplementary Note S.2.4 and Supplementary Figures S15–S18 for analytical details).

The measurements were repeated in commercial reconstituted lyophilised human urine to test the applicability of this sensor in a more complex biological media, especially at clinically relevant levels (between 0.5 × 10^−7^ M and 1 × 10^−6^ M). Signals from CB[7] are visible in the SERS spectra even when the AuNPs were redispersed in urine (Figure 4b) and the presence of all three uretic neurotransmitters could be readily detected using the trained ANN. The predicted amount of catecholamines present in the commercial sample was within the expected range specified by the supplier. Furthermore, when these urine samples were spiked with the three neurotransmitters, the increase in their respective concentrations could readily be quantified by PLSR (Figure 4d, Supplementary Figure S21). These results highlight the robustness and applicability of this system in complex media, even at normal biological concentrations.

## Discussion

It is important to note that the calibration of the system effectively eliminates the influence of the differences in binding affinities of the analytes towards CB[7] on the final results. The system is entirely based on dynamic supramolecular interactions, i.e. assembly process of nanoparticles and guest inclusion inside CB[7], which accounts for some degree of inter and intra-assay variability. Despite the variability, however, the results are reproducible and only limited by the lowest training value of the calibration range. It is also worth mentioning that the errors could likely be further reduced by using more accurate sampling systems (i.e. autosamplers) as well as using a higher number of repeat samples for calibrating the system. Here, the samples were prepared by a single researcher through serial dilutions, using eppendorf micropipettes with different volume ranges and an average of three repeat samples were measured. Automation will tremendously reduce the time required for preparing samples accurately for calibration, but would not increase the time required for analysis of each sample.

To conclude, the absolute determination of analyte levels at low concentrations is usually challenging using colloidal SERS substrates, especially in multiplexed analysis. Thus, this system represents the first SERS-based supramolecular system with multiplexing abilities in aqueous media. The CB[*n*] gold nanoparticle system is solely based upon self-assembly and does not require expensive and time-consuming preparation or storage of specialised SERS substrates, pre-functionalisation, or separation steps. Given the versatility of CB[7] binding to a range of guest molecules, the multiplexing abilities of this technique can be expanded well beyond biological applications. Its potential portability, fast processing times (in under 10 minutes), simplicity and low cost make it a particularly attractive strategy. The method developed in this work can be automated with ease for high throughput analyses and could have tremendous impact on a broad range of applications.

## Methods

All starting materials were purchased from Alfa Aesar and Sigma Aldrich and used as received unless stated otherwise. CB[7] and CB[8] were synthesized according to literature methods^33^. Millipore 18 MΩ.cm H_2_O was used in all experiments unless stated otherwise. Standard stock solutions of all neurotransmitters were freshly prepared prior to analysis. 60 nm citrate-stabilised gold nanoparticles were purchased from British Biocell International. Lyophilised urine samples for catecholamines (Calibrator Lot No. 150 and Control Level II Lot No. 230) were obtained from RECIPE ClinChek-Control. The lyophilised urine samples were reconstituted in dilute hydrochloric acid as specified by the supplier.

### Nuclear magnetic resonance spectroscopy

^1^H NMR and DOSY spectra were recorded on a Bruker Avance 500 BB-ATM (500 MHz) spectrometer. DOSY experiments were carried out using a modified version of the Bruker sequence ledbpgp2s. Spectra were recorded in heavy water (D_2_O) at 298 K. The concentration of CB[7] was fixed at 1 × 10^−3^ M for all the samples. The experiments were processed with standard Bruker 1D and 2D DOSY software. The diffusion coefficients were determined by fitting the intensity decays to equation 1. 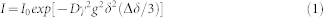
where, I and I_0_ represent the signal intensities in the presence and absence of gradient pulses respectively, D is the diffusion coefficient, *γ* is the ^1^H gyromagnetic ratio, *δ* is duration of the gradient pulse, Δ is the total diffusion time and *g* is the applied gradient strength.

For the 1D titration studies, a series of of solutions were prepared by adding calculated volumes of 4 mM of stock solutions of the guest molecules in D_2_O into 2 mM solution of CB[7], also in D_2_O. The spectra was processed using Mestronova NMR processing software.

### Isothermal titration calorimetry

Isothermal titration experiments were carried out on a NanoITC (TA Instruments) at 25°C in water. The binding equilibria was studied using a cellular CB[7] concentration of 0.1 mM (950 *μ*L) to which a 10-times higher concentrated guest solution was titrated. Typically 25 consecutive injections of 10 *μ*L each were used. All solutions were degassed for at least 15 minutes prior to titration. Heats of dilution were determined by titration of the guest solution into water. The first data point was removed from the data set prior to curve fitting. The data was analyzed with the inbuilt software (NanoAnalyse) with the ‘independent sites' model.

### Surface-enhanced Raman spectroscopy

Raman and SERS spectra were acquired using a 785 nm laser (17.5 mW) and recorded with an Ocean Optics QE65000 Spectrometer. Acquisition time for each spectra was 10 seconds. 200 *μ*L of the 60 nm gold nanoparticle solution was added to a 10 *μ*L pre-mixed CB[7] and neurotransmitter solution (prepared at higher concentrations to make up the final concentrations as required). For analysis of urine samples, CB[7] and neurotransmitters were added to make up the required final concentration. 200 *μ*L gold nanoparticle colloids were centrifuged at 12000 rpm for 60 seconds and 190 *μ*L of supernatant was discarded before addition to 200 *μ*L of urine sample. The extra centrifugation step was carried out in order to maintain the same number of nanoparticles as used in the water samples.

### Multivariate analysis

Multivariate data analysis was performed in Matlab (version 8.1). Artificial Neural Networks analysis was carried out using OXLearn, a matlab-based package^34^. The implemented algorithms contained in the Statistics Toolbox (version 8.2) were used to perform Partial Least Squares Regression. Matlab scripts written by ourselves were used to pre- and postprocess the data (additional information available in Supplementary Note S3).

## Author Contributions

S.K. designed and performed experiments, prepared figures, analysed data and wrote the paper; L.O.H. analysed data; J.d.B. designed and performed DOSY ^1^H-NMR experiments; J.J.B. and O.A.S. supervised through the experimental design, data analyses and writing of the paper.

## Supplementary Material

Supplementary Information Supplementary Information: Quantitative multiplexing with nano-self-assemblies in SERS
