# Supplementary material for: Quantitative multiplexing with nano-self-assemblies in SERS
Source: Sci Rep. 2014 Oct 30;4:6785. doi: 10.1038/srep06785 (PMC4213794; doi:10.1038/srep06785)
Supplement: Supplementary Information — Quantitative multiplexing with nano-self-assemblies in SERS [file srep06785-s1.pdf]

Supplementary Information: Quantitative multiplexing with nano-self-assemblies in SERS

Setu Kasera[a], Lars O. Herrmann[b], Jesús del Barrio[a], Jeremy J. Baumberg[b], and Oren A. Scherman*[a]

[a]Melville Laboratory for Polymer Synthesis, Department of Chemistry, University of Cambridge, CB2 1EW

[b]Nanophotonics Centre, Cavendish Laboratory, University of Cambridge, CB3 0DY

*Email: oas23@cam.ac.uk

August 13, 2014

Contents

S.1 Binding measurements for cucurbit[7]uril and neurotransmitters S2
S.1.1 Diffusion Ordered ^{1}H NMR Spectroscopy . . . S2
S.1.2 Acid dissociation constants (pK_a) of the neurotransmitters . . . S3
S.1.3 Binding Stoichiometries: ^{1}H-NMR Spectroscopy . . . S3
S.1.4 Binding affinities: Isothermal Titration Calorimetry . . . S7
S.1.5 Binding behaviour in mixtures: DOSY ^{1}H-NMR . . . S8
S.1.6 Geometry optimised structures . . . S9

S.2 Stability of SERS signals intensities S11

S.3 SERS spectral analysis S12
S.3.1 SERS spectra of CB[7] with individual neurotransmitters . . . S12
S.3.2 Prominent visible trends with varied concentrations . . . S14

S.4 SERS Data mining S16
S.4.1 Artificial Neural Networks: Measuring the presence or absence of analytes . . . S16
S.4.2 Partial least squares regression . . . S17
S.4.3 Model validation . . . S18
S.4.3.1 R^2 and Q^2 values . . . S18
S.4.3.2 Cross validation . . . S19
S.4.3.3 Validation with independent test sets . . . S21

S.5 Control study with NaCl S22
S.5.1 Validation with independent test sets . . . S22

S.6 Studies in urine with CB[7] S25

S.1 Binding measurements for cucurbit[7]uril and neurotransmitters

S.1.1 Diffusion Ordered ^{1}H NMR Spectroscopy

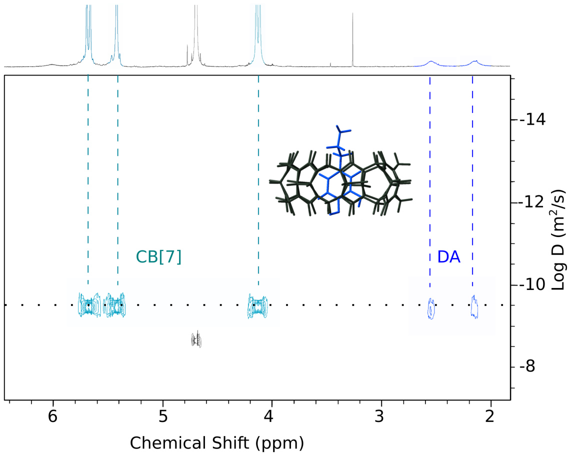

(a)

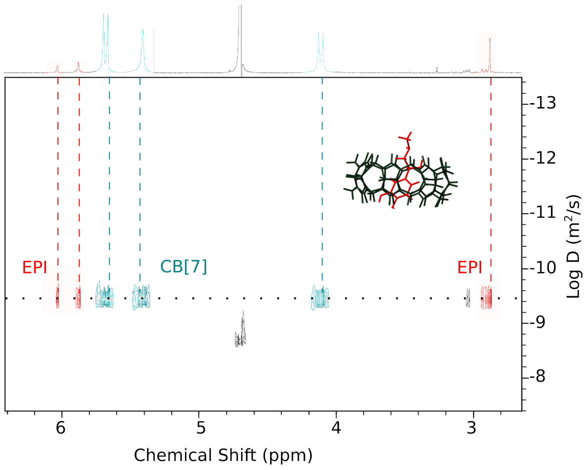

(b)

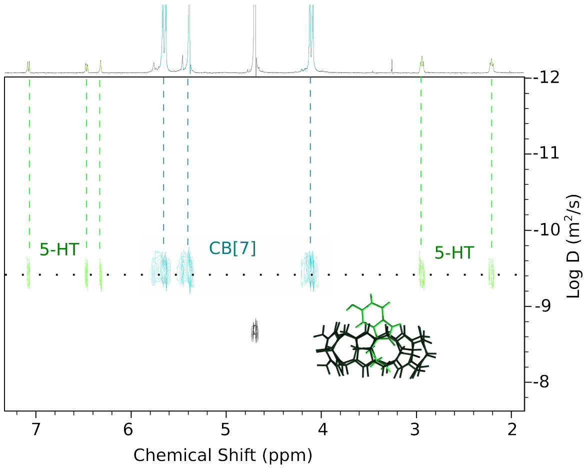

(c)

Figure S1: DOSY ^{1}H NMR spectra for neurotransmitters (a) dopamine, (b) epinephrine and (c) serotonin with CB[7]. The encapsulation of the neurotransmitters (guests) inside the cavity of the heavier macrocyclic host molecule CB[7] results in the observation of a single diffusion coefficient, which is attributed to the host-guest complexes. Binding is observed for all the three neurotransmitters.

S.1.2 Acid dissociation constants (pK_a) of the neurotransmitters

Table S1: pK_a values of dopamine, epinephrine and serotonin at 25 ° C[1]

Neurotransmitter	pK_{a1}	pK_{a2}
Dopamine	8.9	10.6
Epinephrine	8.66	9.95
Serotonin	9.8	11.1

The pK_a values of the labile protons in all three analytes are above the pH of water i.e. pH 7 at 25° C. The order of deprotonation is expected to be phenol (pK_{a1}) followed by the ethylamine group (pK_a2)[2]. Therefore, more than 50% of the respective protonation sites, particularly the ethylamine group, are expected to be protonated in neutral water, in which the analyses were carried out. The charge on the amine functional groups of the alkyl arms are expected to have a stabilising effect on the complex formation through electrostatic interactions with the electronegative carbonyl portals.

S.1.3 Binding Stoichiometries: ^{1}H-NMR Spectroscopy

The upfield shift of the ^{1}H NMR signals of EPI, DA and 5HT guest molecules indicate an inclusion of the three neurotransmitters inside the CB[7] cavity. The degree of shift differs for different protons of the guest as a result of the different degrees of inclusion of the guest inside the shielding environment of the CB[7] cavity and proximities of the protons to the deshielding carbonyl portals. A downfield shift is observed only for the protons of the methyl moiety of the secondary amine of EPI, which suggests that the aliphatic group is spatially closer to the deshielding carbonyl portal region.The inward breathing modes of the macrocycle are slightly restricted on the inclusion of any guest inside its cavity. This leads to the shielding of the CB[7] protons from each other, which is observed as an upfield shift of the CB[7] protons in the NMR spectra.

The titration studies show that all the three monoamines bind to CB[7] with a 1:1 stoichiometry. This is expected as the cavity volume of CB[7] is known to be able to accommodate only one aromatic compound at any time. The results obtained by Isothermal Titration Calorimetry (Section S.1.4) support the 1:1 stoichiometries obtained for all CB[7]-neurotransmitter complexes.

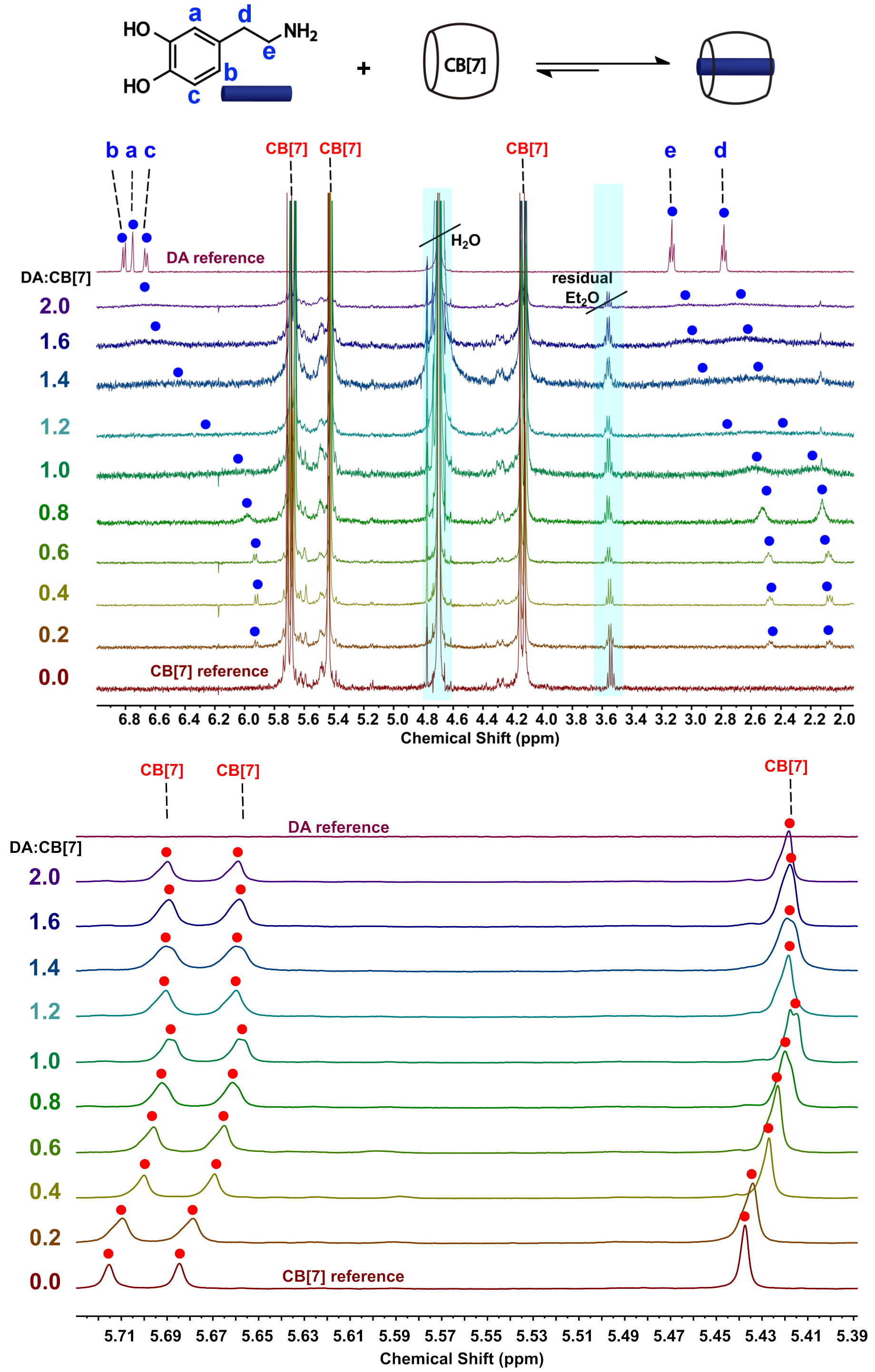

Figure S2: Titration of dopamine into CB[7] studied by ^{1}H NMR in D_2O.

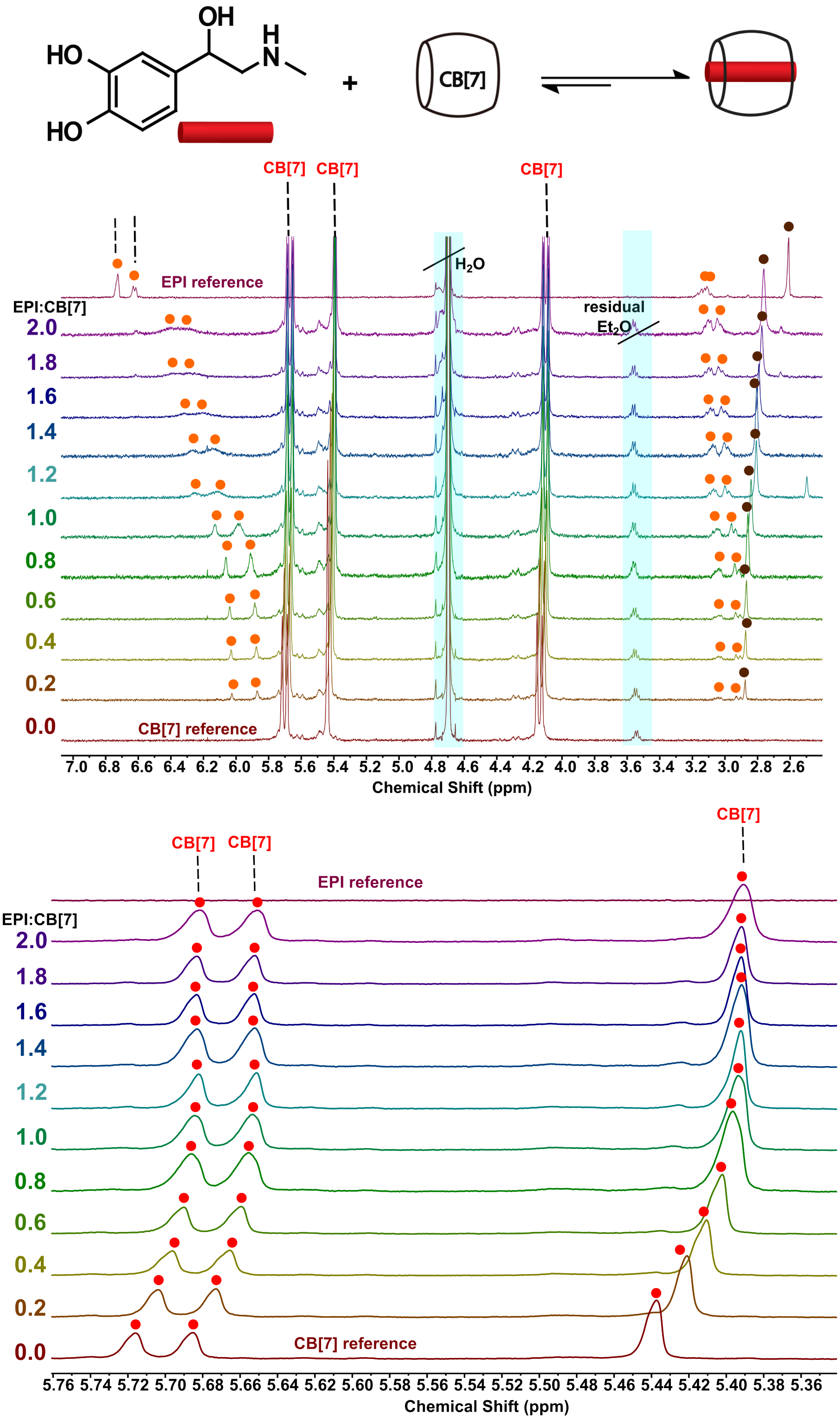

Figure S3: Titration of epinephrine into CB[7] studied by ^{1}H NMR in D_2O.

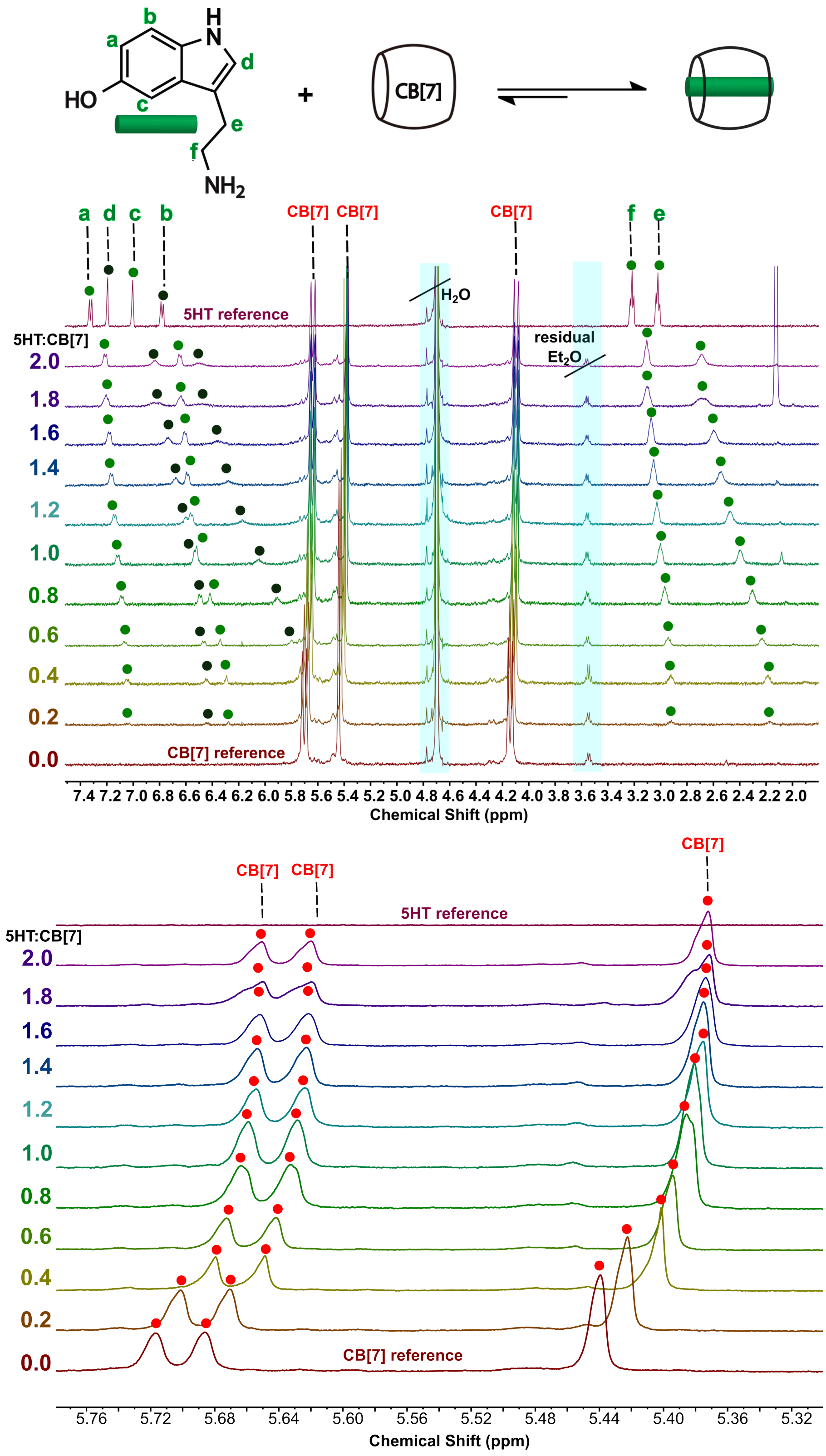

Figure S4: Titration of serotonin into CB[7] studied by ^{1}H NMR in D_2O.

S.1.4 Binding affinities: Isothermal Titration Calorimetry

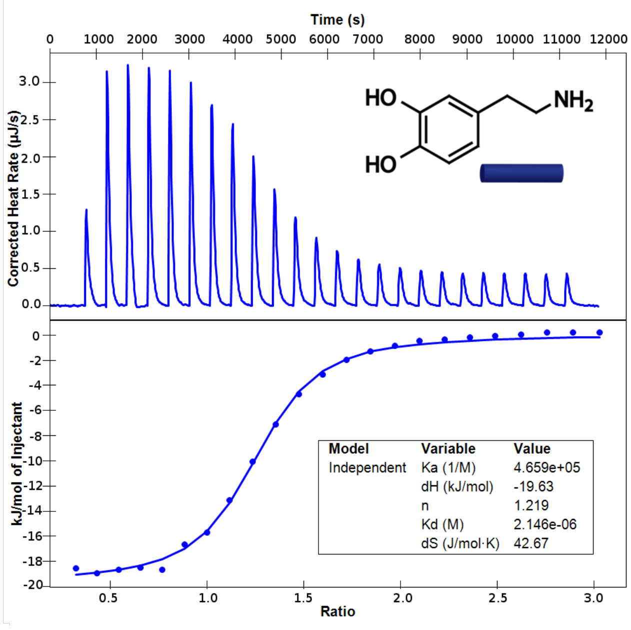

(a) Dopamine

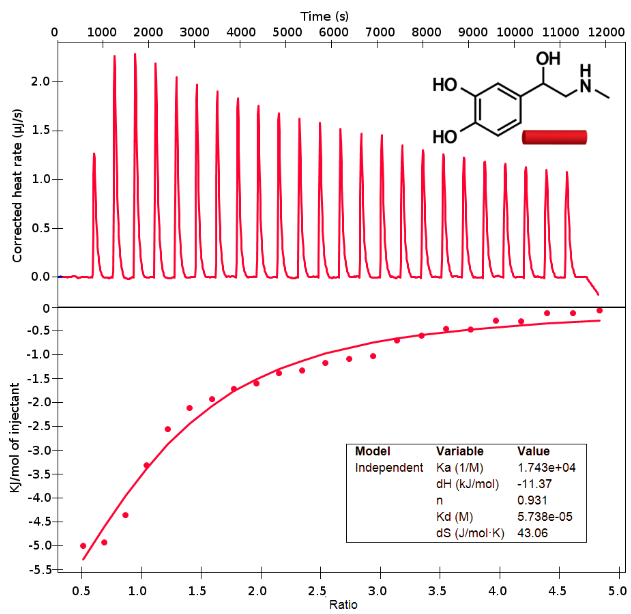

(b) Epinephrine

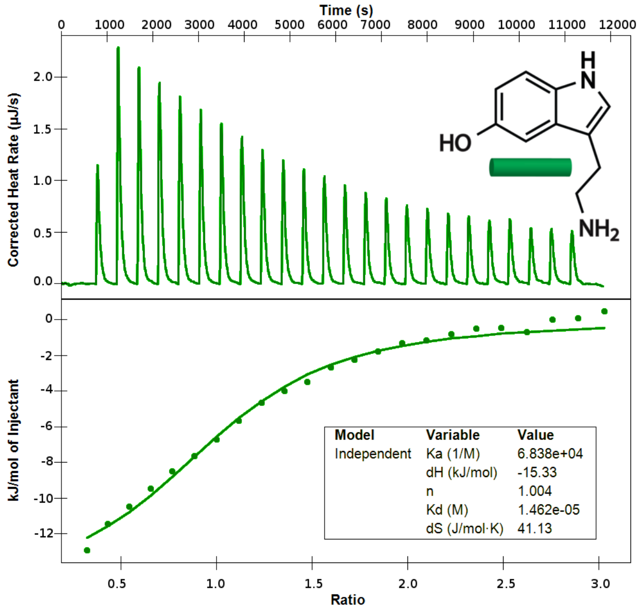

(c) Serotonin

Figure S5: Binding isotherms of CB[7] with the three neurotransmitters in H_2O studied by Isothermal Titration Calorimetry. All three neurotransmitters bind to CB[7] with similar affinities and show comparable enthalpic and entropic gains for the host-guest complex formation.

S.1.5 Binding behaviour in mixtures: DOSY ^{1}H-NMR

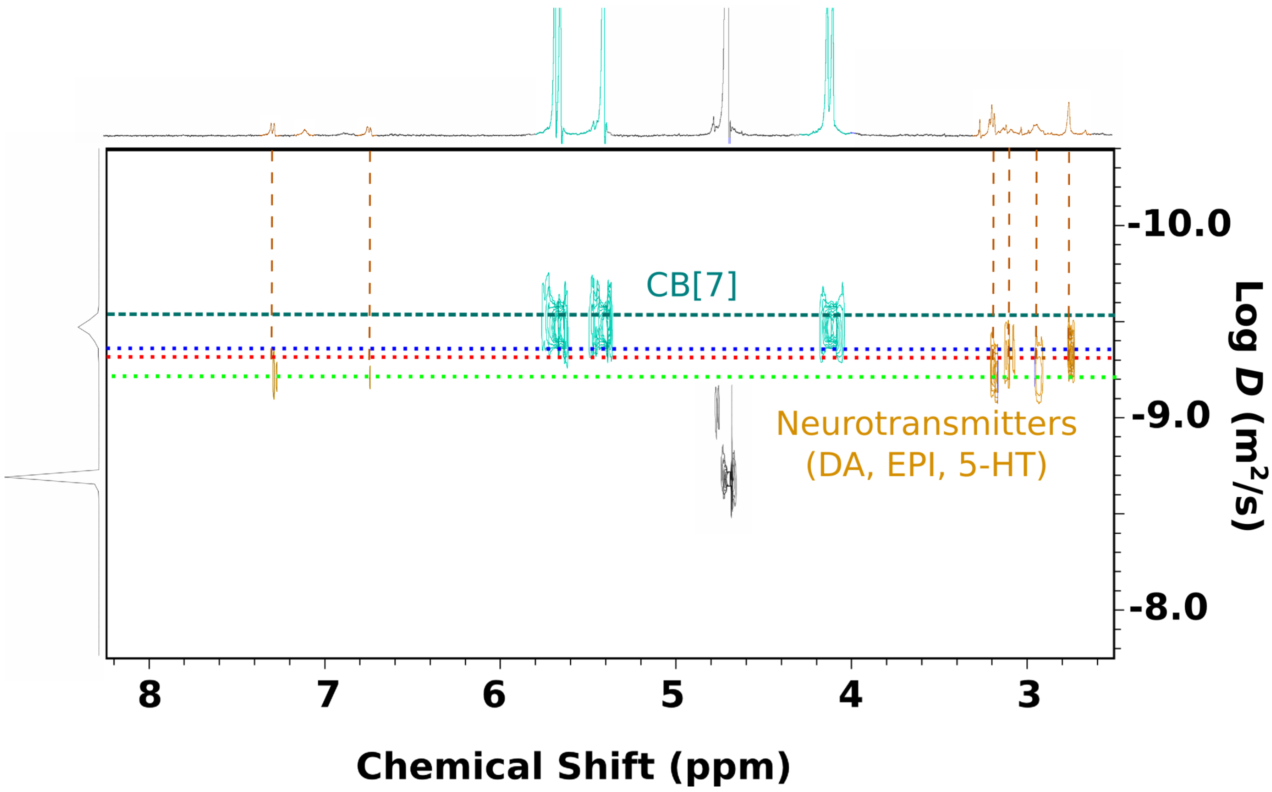

Figure S6: Equimolar mixture of CB[7]:EPI:DA:5HT. In the absence of sufficient concentration of CB[7] for all three neurotransmitters, the guests compete for the host with similar affinities, which results in unbound neurotransmitters in the solution. This is indicated by the larger diffusion coefficient values for the neurotransmitters and a smaller value for CB[7].

S.1.6 Geometry optimised structures

The starting geometry of the neurotransmitters were built invoking standard bond lengths and bond angles using the Avogadro Model Builder followed by step-wise geometry optimizations on HF/3-21G using GAMESS[3]. The CB[7] starting geometry was retrieved from the Cambridge Structural database[4] (cucurbit[7]uril, refcode FUYHIR). The nature of the stationary points on the potential energy surface were evaluated by calculation of the full Hessian matrix and vibrational frequencies (visualised with MacMolPlt[5]). No negative elements of Hessian matrix and no imaginary frequencies confirmed that the obtained structures were the minima on the potential energy surfaces. The geometries were further optimised at the DFT-B3LYP/6-31G* level of theory. Representative geometries of the host guest complexes are shown in Figure S7. All the above calculation were carried out in the gas phase. Calculations in aqueous phase are ongoing in our research group. The calculations were performed using the Darwin Supercomputer of the University of Cambridge High Performance Computing Service (http://www.hpc.cam.ac.uk/), provided by Dell Inc. using Strategic Research Infrastructure Funding from the Higher Education Funding Council for England and funding from the Science and Technology Facilities Council.

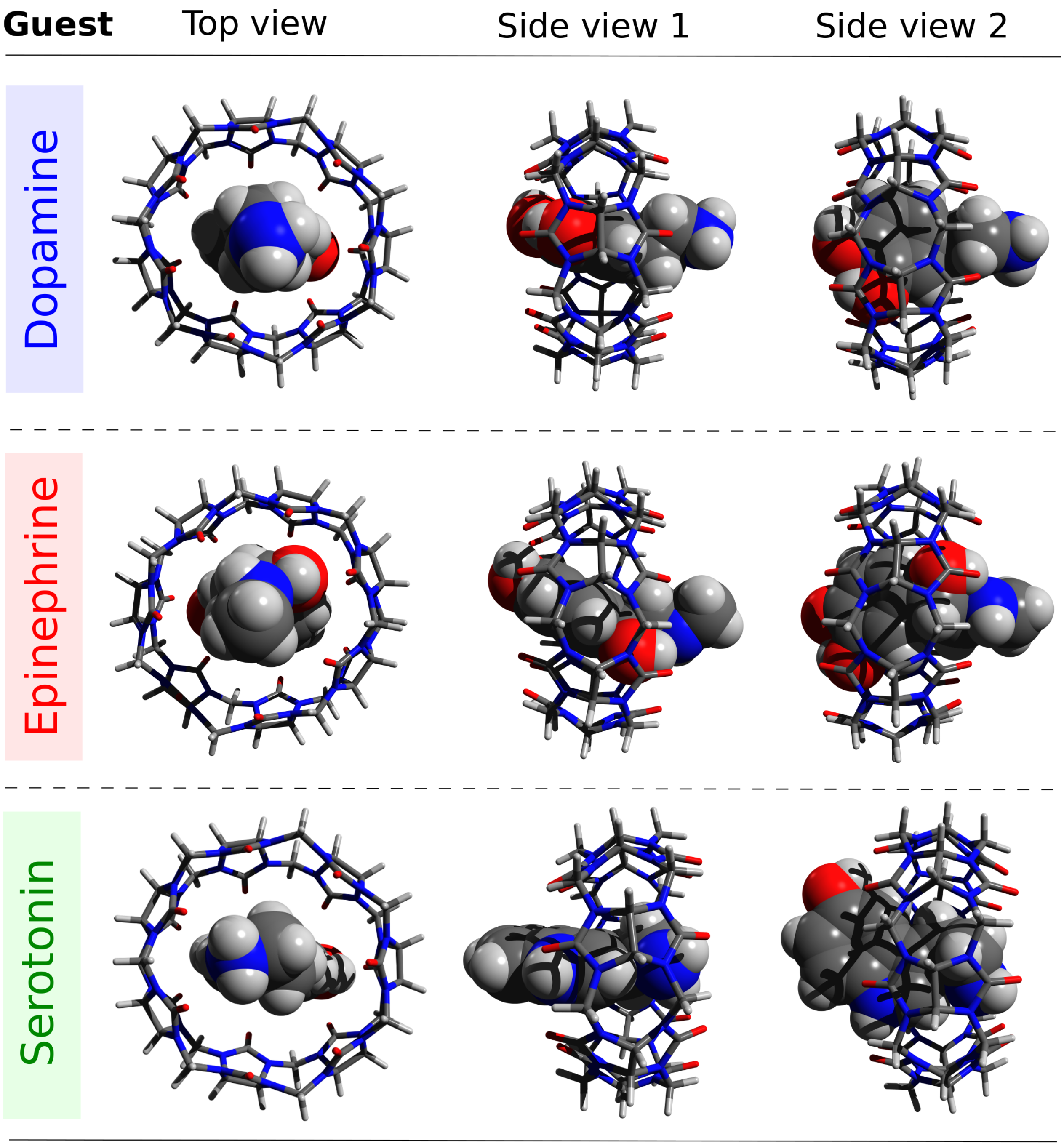

Figure S7: Representative CB[7] host-guest complex geometries with dopamine, epinephrine and serotonin geometry optimised at DFT-B3LYP/6-31G* level of theory in gas phase.

S.2 Stability of SERS intensities

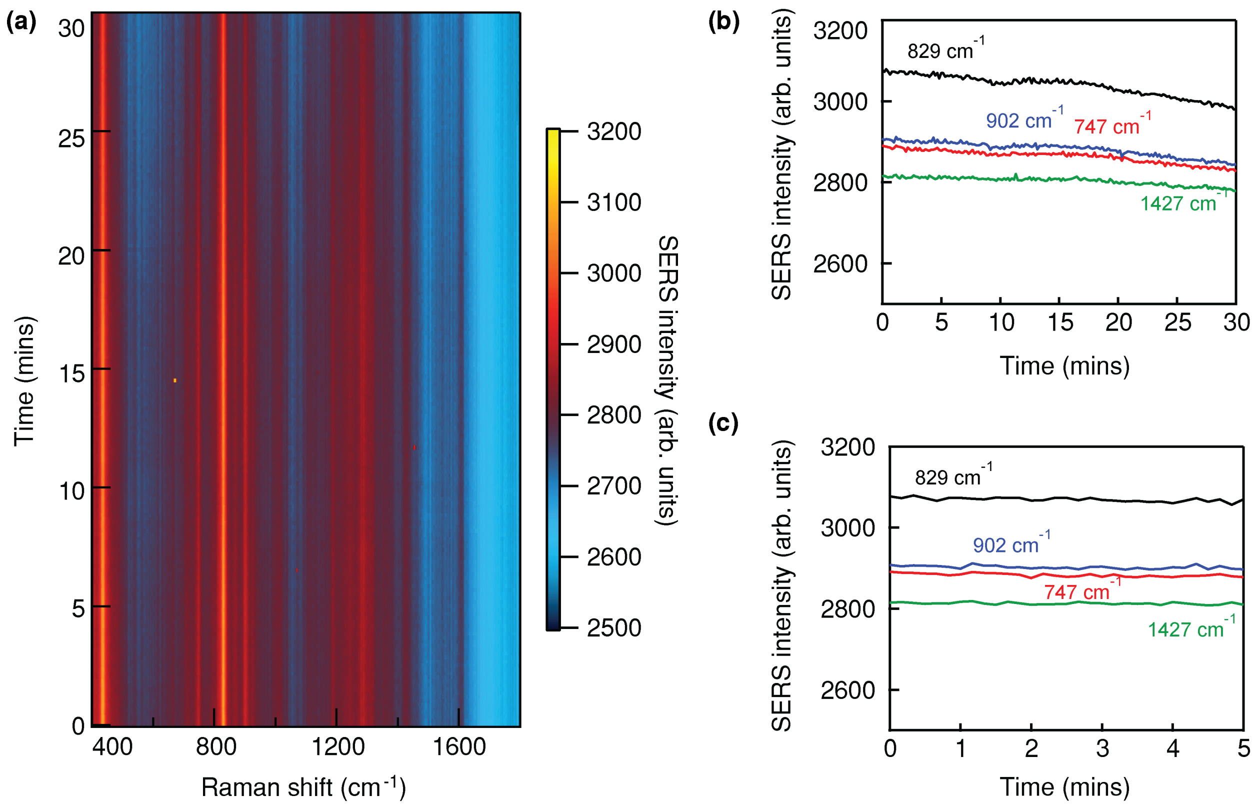

Figure S8: (a) Temporal dependence of SERS spectra collected over 30 mins, where CB[7] was added at time t=0 (Laser power, 17.5 mW; excitation wavelength, 785 nm; resolution, $\sim$ 10 cm^{-1}; integration time per spectrum, 10 s). (b) Temporal function of SERS intensities at selected peak positions. The slight decrease in intensities indicates precipitation of larger aggregates over time. (c) Zoom-in region of (b) over the first 5 minutes shows good stability in SERS intensities (<0.6% relative standard deviation), eliminating the need to take measurements at precise time points after aggregation has begun.

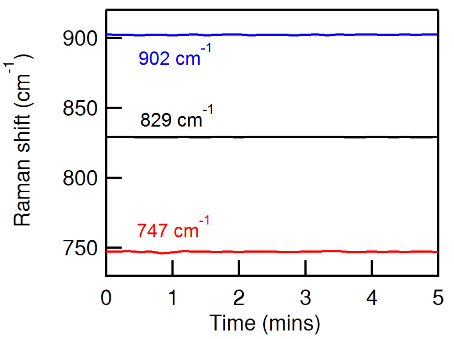

Figure S9: Temporal function of SERS signal position shows good stability with a relative standard deviation of <0.05% and a maximum absolute deviation of 1.4 cm^{-1} over 30 mins.

S.3 SERS spectral analysis

S.3.1 SERS spectra of CB[7] with individual neurotransmitters

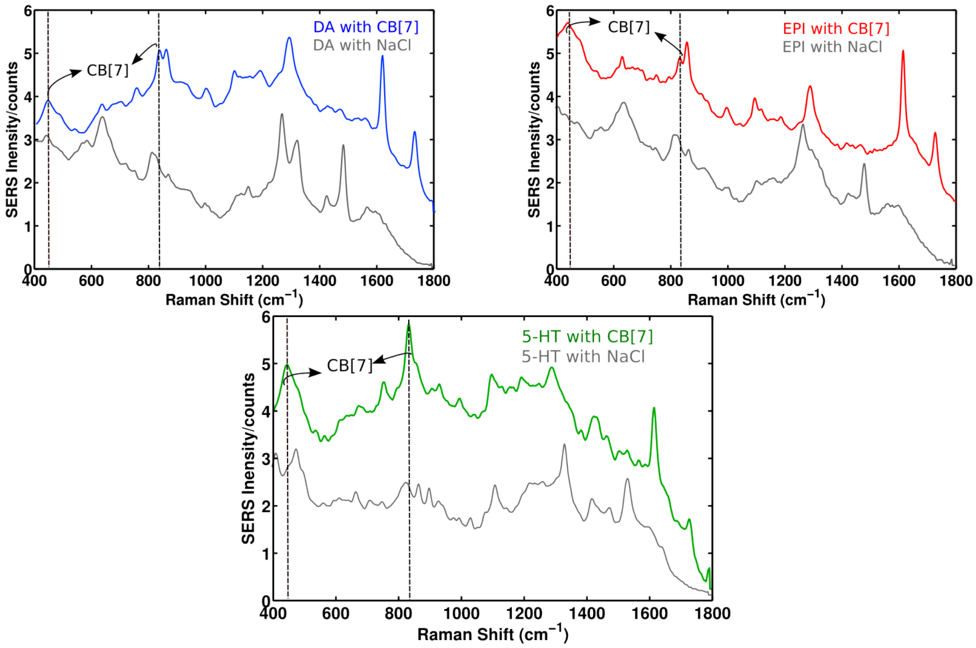

Figure S10: Comparison of representative SERS spectra of the neurotransmitters (a) epinephrine, (b) dopamine and (c) serotonin where salt (black) and CB[7] (coloured) were used as aggregating agents for the gold nanoparticles. Signals from CB[7] (vertical dashed lines) as well as the neurotransmitters are visible in the spectra from the host-guest complex and missing in the absence of CB[7]. In the presence of salt, different vibrational modes of the neurotransmitters are visible. Laser power, 17.5 mW; excitation wavelength, 785 nm; resolution, $\sim$ 10 cm^{-1} laser irradiation time, 10 s.

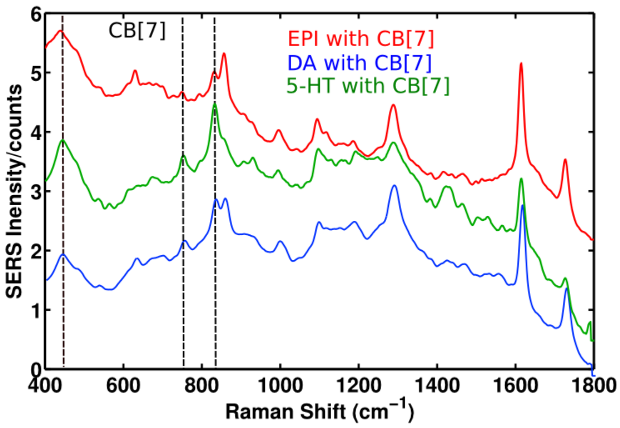

Figure S11: Comparison of SERS spectra of DA.CB[7], EPI.CB[7] and 5-HT.CB[7] in water. Laser power, 17.5 mW; excitation wavelength, 785 nm; resolution, $\sim$ 10 cm^{-1} laser irradiation time, 10 s.

S.3.2 Prominent visible trends with varied concentrations

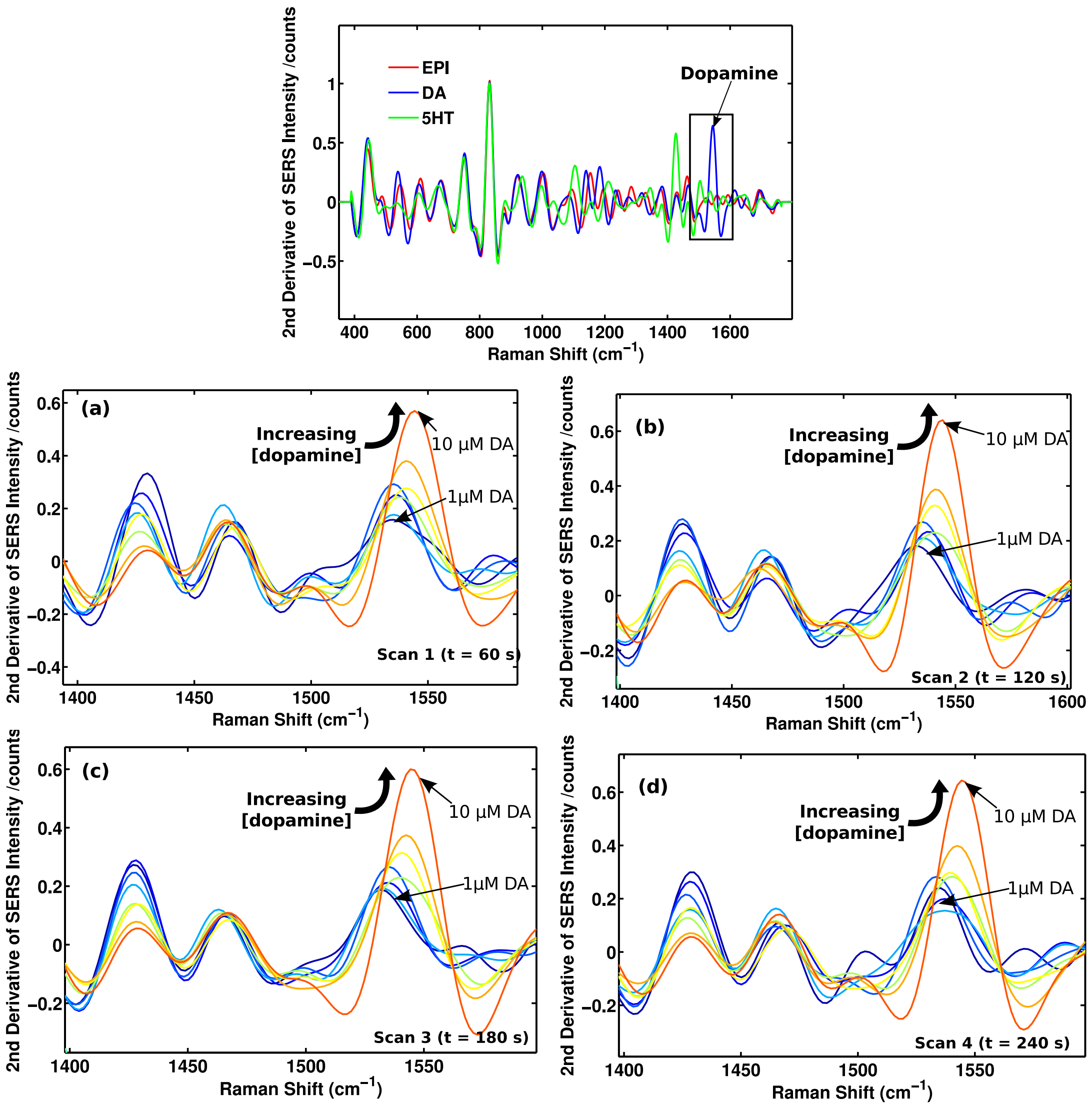

Figure S12: Prominent features visible in the SERS spectra of EPI, DA, 5HT mixtures with CB[7]. **a**, SERS spectra of individual solutions of EPI, DA, 5HT (1×10^{-6} M) highlighting an isolated signal from DA. **b-e**, Zoomed-in spectral region highlighting the signal from DA at ~1550 cm^{-1} shows an increasing trend in peak intensity when the concentration of DA is increased in the mixtures.

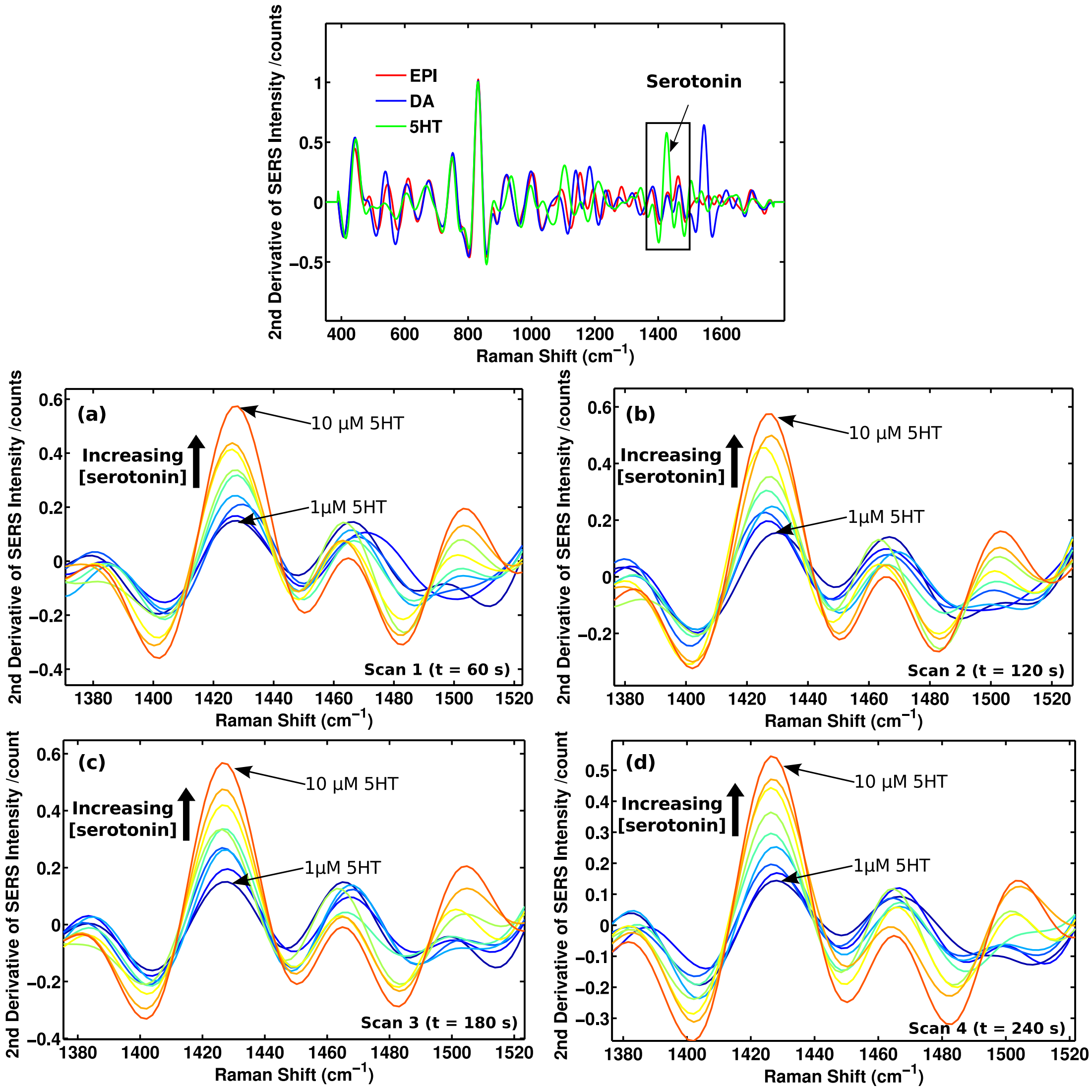

Figure S13: Prominent features visible in the SERS spectra of EPI, DA, 5HT mixtures with CB[7]. **a**, SERS spectra of individual solutions of EPI, DA, 5HT (1×10^{-6} M) highlighting an isolated signal from 5HT. **b-e**, Zoomed-in spectral region highlighting the signal from 5HT at $\sim$1425 cm^{-1} shows an increasing trend in peak intensity when the concentration of 5HT is increased in the mixtures.

S.4 SERS Data mining

S.4.1 Artificial Neural Networks: Measuring the presence or absence of analytes

The principles of Artificial neural networks (ANN) have been discussed elsewhere [6], but in brief, ANN comprise layers of interconnected nodes to form an array. The layers are classified into an input layer, one or more intermediate (or 'hidden') layers and an output layer. All nodes in a given layer are connected to all the nodes in the consequent layer. In this study, the simple three-layer feed-forward network was implemented.

Calibration or 'training' of the ANN requires an input data set and a corresponding output data set. For example, in this study, 715 data points consisting of numerical values of Raman intensities formed the 715 nodes of the input layer. Values of either '0' or '1' were assigned to each of the 3 desired output nodes, where each node represented the result for a single neurotransmitter i.e. '0' for 'absent' and '1' for 'present'.

Inter-node connections are assigned numerical 'weights', which are set to randomly selected values at the start. The weights are adjusted during the training process based on in-built learning patterns programmed in the software.

Several parameters affecting the network architecture and function were investigated by residual analyses to optimise the network. The output pattern generated using the following parameters showed the best match to the target pattern, with 22 out of 24 predictions being correct.

Table S2: Optimised learning parameters for analysis with artificial neural networks

learning	
method	backpropagation
learning rate	0.4
momentum	0.7
training paradigm	
Number of sweeps	15000
presentation order	random with replacement
weights initialisation	
type	random seed
range	-0.18 to +0.18
layers	
size of input layer	715 × 30
size of hidden layer	300 × 1
size of output layer	3 × 1

Table S3: Target and measured values with optimised ANN parameters

	Target pattern			Calculated pattern		
Sample	EPI	DA	HT	EPI	DA	HT
A	1	1	1	1.00	1.00	1.00
B	1	1	1	0.52	0.87	1.00
C	0	1	1	0.00	0.68	1.00
D	1	1	1	0.73	0.98	1.00
E	0	0	1	0.00	0.00	1.00
F	1	1	1	1.00	1.00	1.00
G	1	0	0	0.97	1.00	1.00
H	1	1	1	1.00	1.00	1.00

1=analyte present; 0 = analyte absent.
See Fig. S14 for schematic representation.

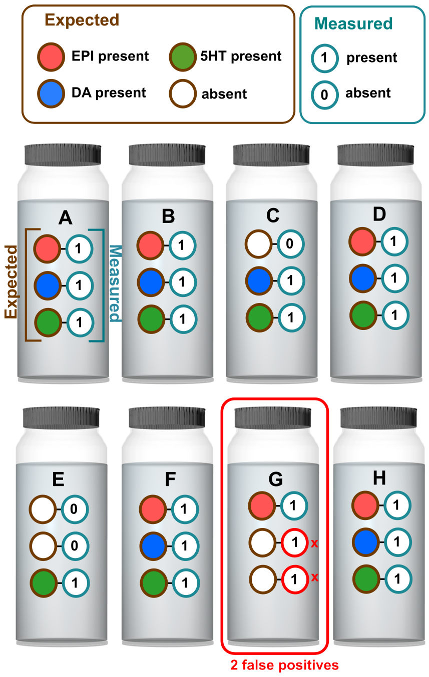

Figure S14: Results of ANN to determine the presence or absence of an analyte in a mixture.

S.4.2 Partial least squares regression

The goal of the algorithm is to create a regression model which can extract the concentrations of p analytes in a mixture from its corresponding SERS spectrum. To build this regression model, the SERS spectra from a set of m mixtures with known concentrations are measured (calibration set). The spectra are stored in a matrix $\mathbf{X}_\mathrm{c}$ where each row of the matrix contains the intensities of each SERS spectrum obtained at n different wavenumbers. Therefore, the dimension of this matrix $\mathbf{X}_\mathrm{c}$ is $m \times n$. Similarly, the corresponding known concentrations of the analytes in each mixture (calibration concentrations) are stored in a $m \times p$ matrix $\mathbf{Y}_\mathrm{c}$. Since n is typically large compared to m, $\mathbf{X}_\mathrm{c}$ is likely singular and ordinary regression is not possible. Here, we make use of partial least squares regression (PLSR), one of the most established methods to cope with such multicollinear data.[8]

Regression model The Matlab implementation of the SIMPLS algorithm[8] was employed to regress the (preprocessed) spectra of the calibration set $\mathbf{X}_\mathrm{calib}$ onto the known corresponding concentrations $\mathbf{Y}_\mathrm{calib}$ of the mixtures. This produces a regression model $\mathbf{B}_\mathrm{PLS}$, which can then be used to predict the concentrations of the test set $\mathbf{Y}_\mathrm{pred} = \mathbf{X}_\mathrm{test}\mathbf{B}_\mathrm{PLS}$. For a detailed description of the algorithms, we refer the reader to relevant literature.[8, 9]

Preprocessing The spectra were truncated in order to eliminate noisy regions close to the edge of the spectral detection band of the spectrometer. After base-line centring and normalisation by area, a standard normal variate transformation was applied and a second-order Savitzky-Golay derivative was taken to reduce the background and high-frequency noise.

S.4.3 Model validation

S.4.3.1 R^2 and Q^2 values

The best validation of a model is the use of a test set (validation set) that contains independent measurements, but other statistical measures can be used to gauge the quality of the model, such as R^2 and Q^2 (cross validated R^2) values. They are calculated as[9]

$$R^2 = 1 - \mathrm{RSS}/\mathrm{SS} \tag{1}$$

$$Q^2 = 1 - \mathrm{PRESS}/\mathrm{SS} \tag{2}$$

with the sum of squares $\mathrm{SS} = \sum_i (y_i - \bar{y})^2$ and the fitted residual sum of squares $\mathrm{RSS} = \sum_i (y_i - f_i)^2$. Here, y_i denotes the measured concentrations, f_i the expected concentrations and $\bar{y}$ the mean of the measured concentrations. The measured residual sum of squares PRESS is calculated as the sum of squares over the resulting measurement errors of a leave-one-out cross validation.

The R^2-value describes how well the model fits the data and the leave-one-out Q^2-value judges the predictive ability of the model. The number of components used in the model is chosen such that both R^2 and Q^2 are high. However, it is important to note that high R^2/Q^2-values are not a sufficient criteria to ensure good predictive abilities of the regression model and cannot replace validation with an independent test.[10]

The percentage of variance in the calibration sets explained by the regression model as a function of the number of used PLS components is shown in Figure S15(a) (for H_2O) and Figure S16(a,b) (for urine), respectively. The higher complexity of the urine data manifests in a larger number of PLS components required to account for the same amount of variance. Clearly visible is how preprocessing successfully reduces noise and interferences, thus enabling reduction of required PLS components.

The R^2/Q^2-values of calibration sets are shown in Figure S15(b) (for H_2O) and Figure S16(c,d) (for urine). Both values are high ($R^2 > 0.95$, $Q^2 > 0.85$) for models with ~ 8 PLS components. The larger values for calibration in H_2O are expected due to the lower complexity of the SERS spectra compared to urine and are in line with the slightly better predictive abilities observed (cf. Figures S18 and S21).

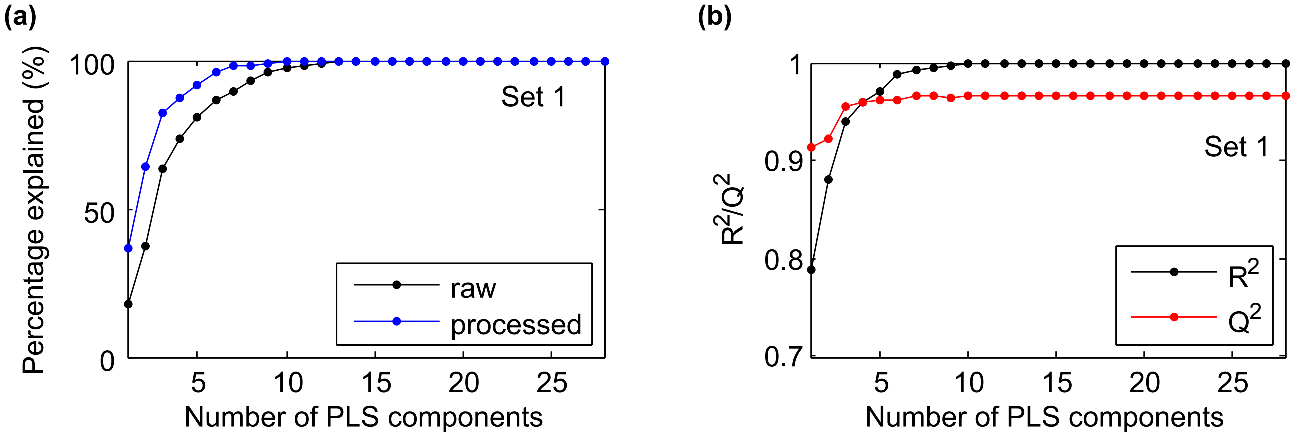

Figure S15: Validation of regression model in H_2O. (a) Percentage of variance of the calibration set explained by the regression model as a function of retained PLS components for raw data and preprocessed data. (b) R^2 and Q^2-values as a function of the number of components in the regression model (for preprocessed).

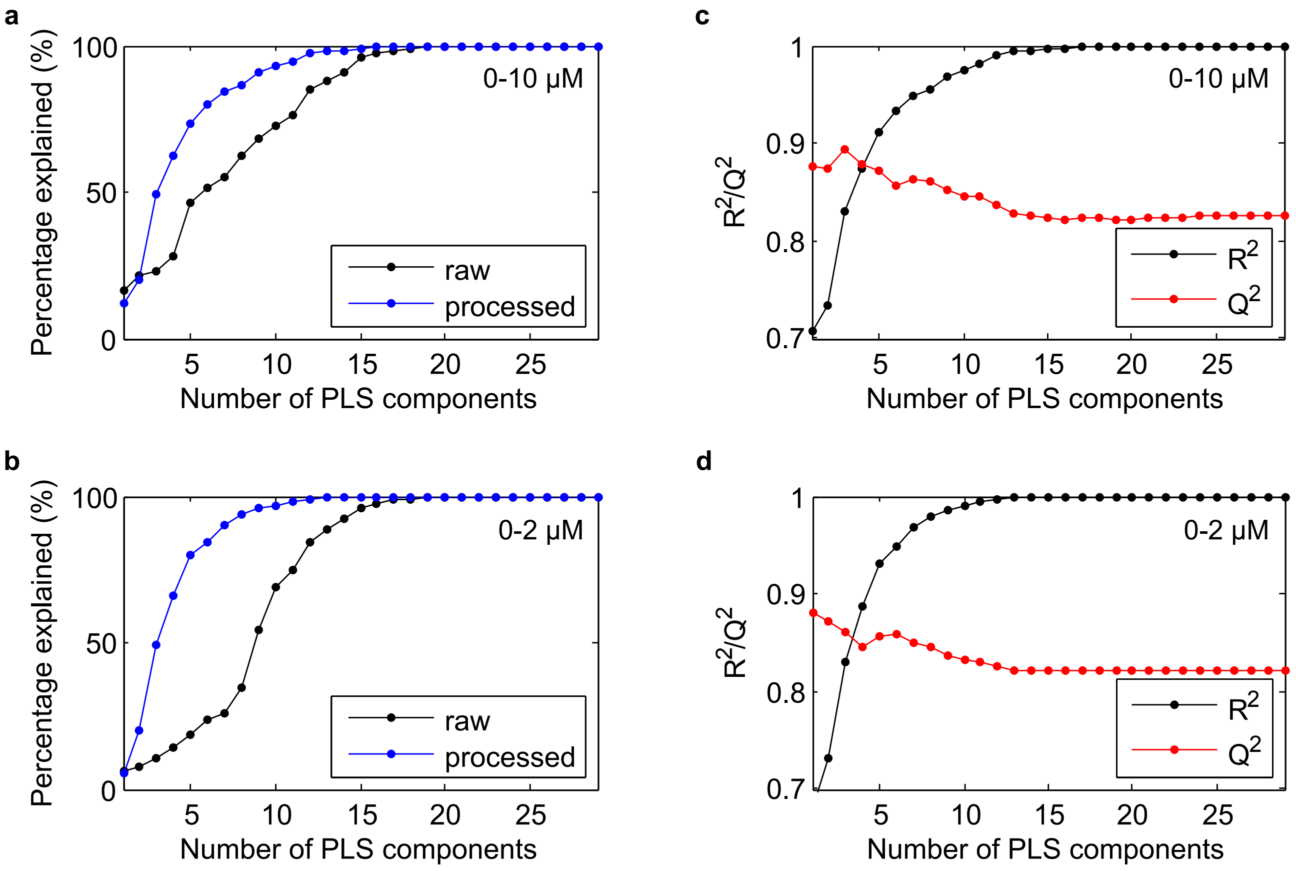

Figure S16: Validation of regression model in urine. Percentage of variance of the calibration set explained by the regression model as a function of retained PLS components for data in urine (a) between 0-10 *mu*M and (b) 0-2 *mu*M (raw and preprocessed). (c,d) Corresponding R^2 and Q^2-values as a function of the number of components in the regression model (for preprocessed data).

S.4.3.2 Cross validation

The calibration data was cross-validated in order to assess and optimise the predictive performance of the calibration model. The results of the cross-validation are shown in Figure S15.

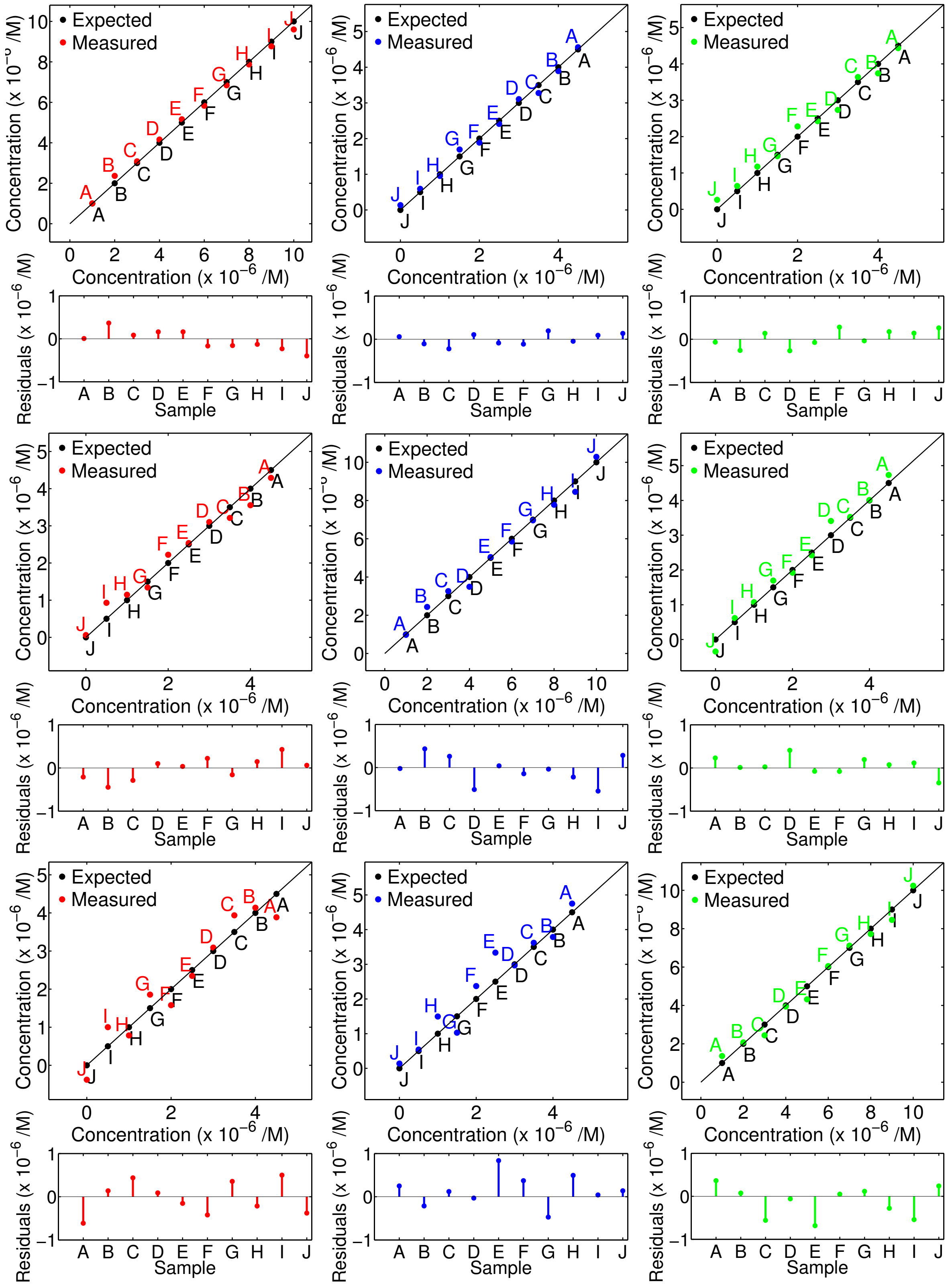

Figure S17: Simple cross-validation of the calibration dataset for every second scan.

S.4.3.3 Validation with independent test sets

As mentioned previously, the best way to evaluate the prediction ability of a calibration dataset is by acquiring an independent set of data, where the concentrations are known. The variability and standard deviation between the expected and measured concentrations using the calibration data will indicate the goodness of the data and its prediction ability for real data.

Eight samples (A to H) containing the mixtures of the three analytes in random concentrations were prepared. The SERS spectra were collected in the same manner as for the calibration dataset of data was collected as an independent 'test' dataset to establish the quality of the collected calibration data. Each set was measured in triplicates to establish a standard deviation error for each data point. The expected and predicted concentrations are given below:

Table S4: Concentrations† of neurotransmitters in individual test sets

	Expected concentrations			Measured concentrations											
				Set1			Set2			Set3			average ± standard deviation		
Sample	EPI	DA	HT	EPI	DA	HT	EPI	DA	HT	EPI	DA	HT	EPI	DA	HT
A	1.0	4.0	5.0	1.4	3.6	5.0	0.5	4.3	5.2	1.1	3.2	5.6	1.0 ± 0.4	3.7 ± 0.5	5.3 ± 0.3
B	3.0	2.0	5.0	2.6	2.1	5.3	3.5	2.5	4.0	2.2	2.2	5.6	2.8 ± 0.7	2.2 ± 0.2	5.0 ± 0.9
C	2.0	3.0	5.0	1.2	2.9	5.9	1.3	3.0	5.6	1.9	2.2	5.9	1.5 ± 0.4	2.7 ± 0.4	5.8 ± 0.2
D	0.5	1.5	8.0	0.2	1.9	7.8	0.3	1.1	8.6	0.2	2.2	7.6	0.2 ± 0.04	1.7 ± 0.6	8.0 ± 0.5
E	1.0	6.0	2.0	1.1	6.1	2.8	1.9	5.9	2.2	1.6	5.5	2.9	1.5 ± 0.4	5.8 ± 0.3	2.6 ± 0.4
F	7.0	1.5	1.5	7.2	1.2	1.6	6.5	1.2	2.3	7.6	1.5	0.9	7.1 ± 0.6	1.3 ± 0.2	1.6 ± 0.7
G	4.0	2.0	4.0	4.5	1.3	4.2	4.4	2.0	3.6	4.7	1.1	4.2	4.5 ± 0.2	1.5 ± 0.5	4.0 ± 0.3
H	3.5	3.5	3.0	3.5	2.8	3.6	2.8	3.4	3.8	3.2	3.0	3.8	3.2 ± 0.4	3.1 ± 0.3	3.7 ± 0.1

†*values are* $\times 10^{-6}$ mol L^{-1}

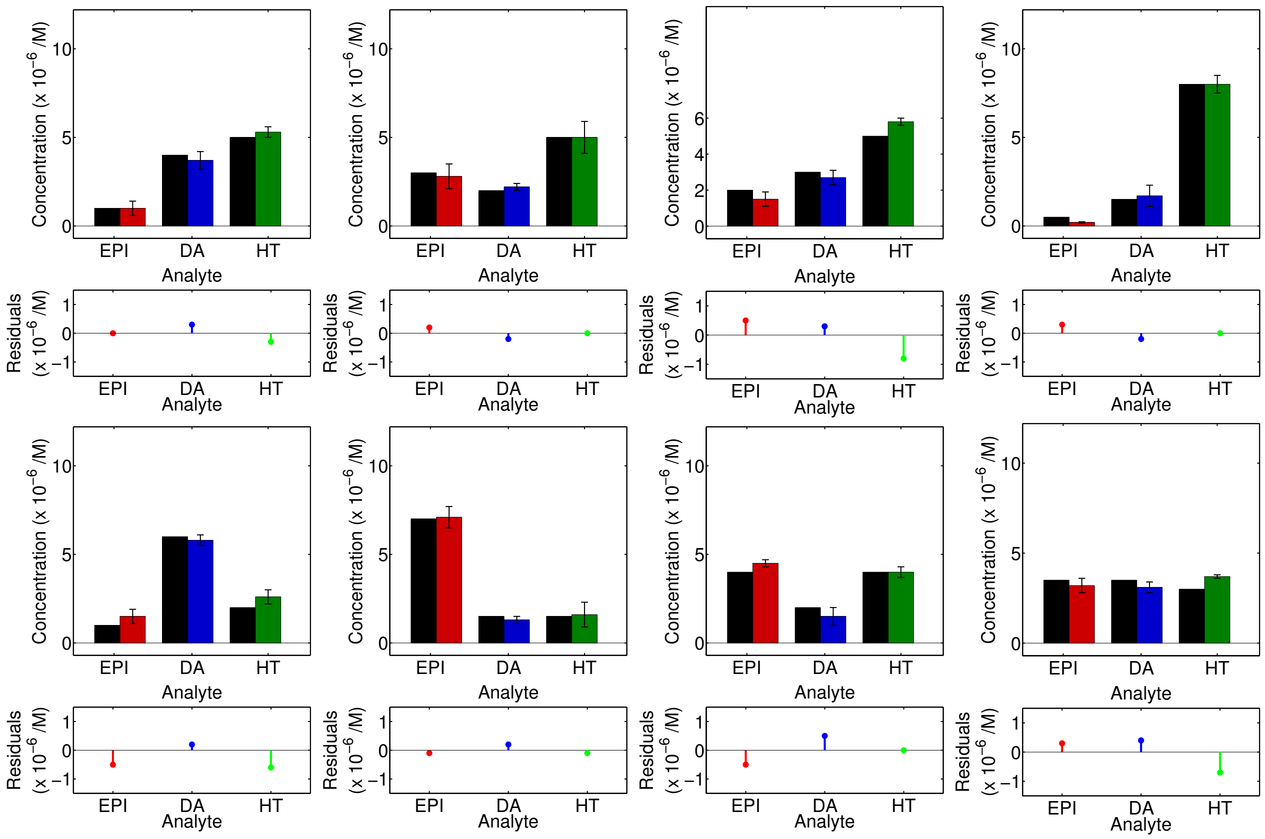

Figure S18: Independent validation of the calibration dataset with test samples A-J containing randomly chosen concentrations of the three neurotransmitters.

S.5 Control study with NaCl

Samples were prepared in the same concentrations as those chosen for the calibration with CB[7]. All analysis for the calibration were carried out identically as for CB[7].

S.5.1 Validation with independent test sets

Given the effectiveness of the calibration set, ten samples (A to E) were prepared containing the mixtures of the three neurotransmitter analytes in random concentrations. Since the analytes have different number of functional groups, they could compete for the nanoparticle surface directly in the absence of CB[7] and this might then distort their population in the media. The SERS spectra were collected in the same manner as for the calibration dataset as an independent 'test' dataset to establish the quality of the collected calibration data. Each set was measured in triplicates to establish a standard deviation error for each data point. The expected and predicted concentrations are given below:

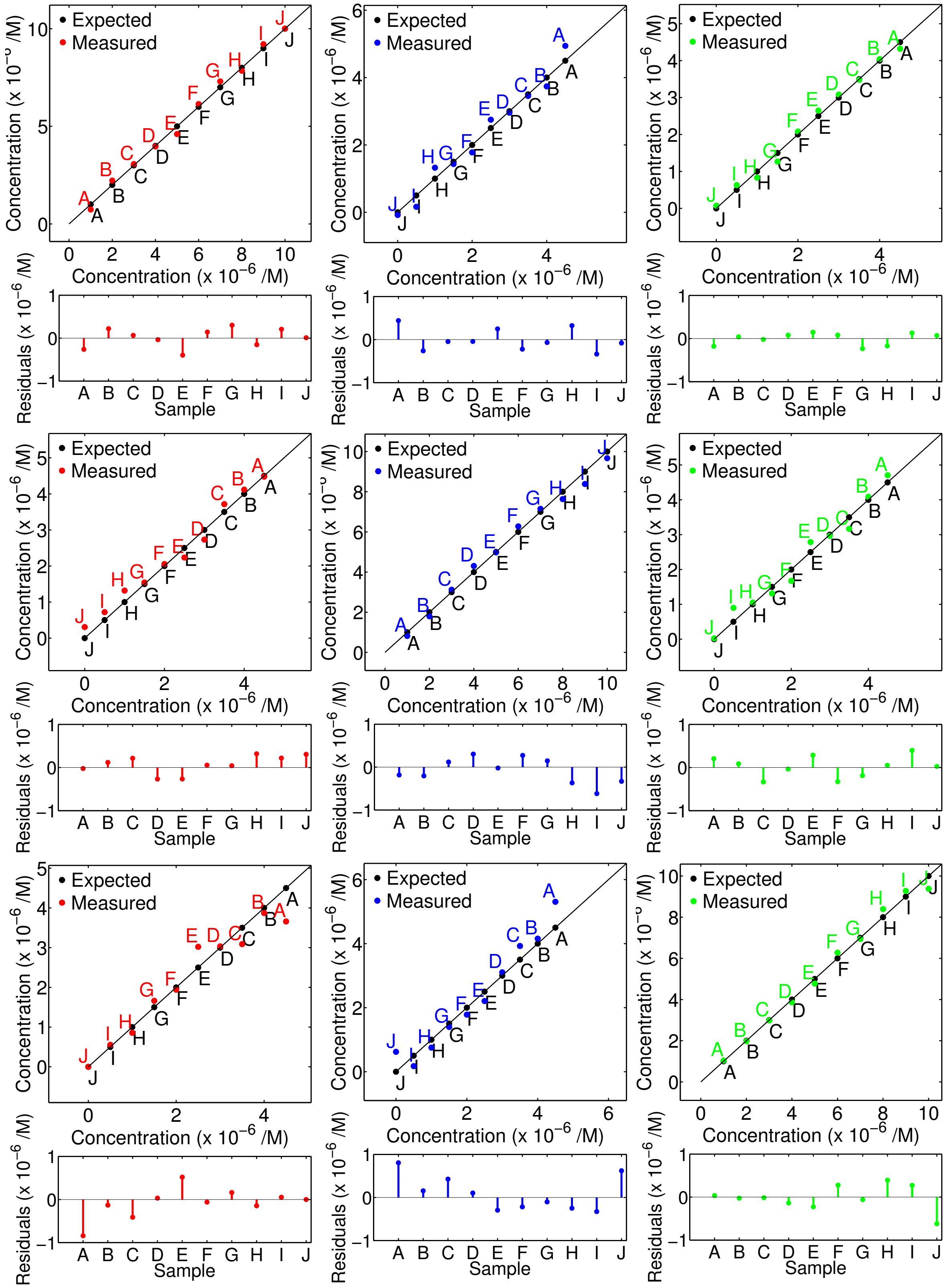

Figure S19: Simple cross-validation of the calibration dataset for every second scan.

Table S5: Concentrations† of neurotransmitters in individual test sets (with NaCl)

	Expected concentrations			Measured concentrations											
				Set1			Set2			Set3			average ± standard deviation		
Sample	EPI	DA	HT	EPI	DA	HT	EPI	DA	HT	EPI	DA	HT	EPI	DA	HT
A	6.0	9.0	2.0	3.0	2.4	4.6	3.1	0.9	5.9	2.5	2.9	4.6	2.9 ± 0.3	2.1 ± 1.0	5.1 ± 0.8
B	9.0	8.0	1.0	5.3	3.0	1.7	2.4	3.3	4.3	4.2	4.4	1.5	3.9 ± 1.5	3.6 ± 0.7	2.5 ± 1.6
C	5.0	2.0	0.0	6.5	2.9	0.6	5.7	3.2	1.1	6.6	2.7	0.7	6.3 ± 0.4	2.8 ± 0.1	0.8 ± 0.2
D	0.0	7.0	9.0	-0.2	4.2	6.1	-1.2	5.0	6.1	-0.9	4.7	6.1	-0.8 ± 0.5	4.7 ± 0.4	6.1 ± 0.0
E	3.0	3.0	3.0	0.5	3.2	6.3	-0.2	3.4	6.8	1.3	2.4	6.4	0.5 ± 0.7	3.0 ± 0.5	6.5 ± 0.3

†*values are* $\times 10^{-6}$ mol L^{-1}

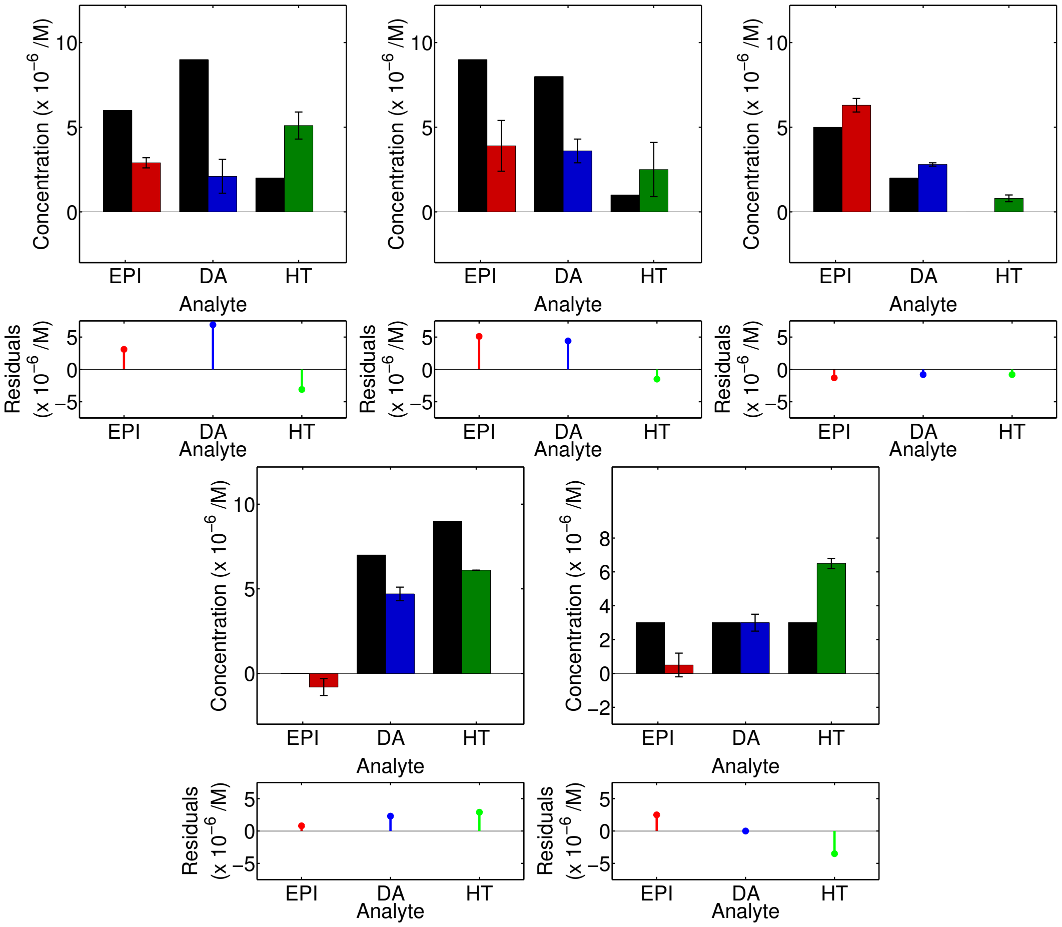

Figure S20: Independent validation of the calibration dataset with test samples with test samples A-J containing randomly chosen concentrations of the three neurotransmitters. NaCl (20 mM) was used as an aggregating agent in this study

S.6 Studies in urine with CB[7]

Table S6: PLS measured results for [EPI], [DA] and [5-HT] for 8 urine samples spiked with randomly chosen amounts of EPI, DA and 5-HT.

	Expected concentrations			Measured concentrations											
				Set 1			Set 2			Set 3			average ± standard deviation		
Sample	EPI	DA	HT	EPI	DA	HT	EPI	DA	HT	EPI	DA	HT	EPI	DA	HT
A	4.66	4.57	2.50	4.86	4.74	1.90	4.45	4.41	2.64	4.00	4.28	3.13	4.44 ± 0.43	4.48 ± 0.24	2.56 ± 0.62
B	2.16	6.57	3.50	2.06	5.15	4.16	1.57	6.00	3.75	2.02	6.02	3.34	1.88 ± 0.27	5.72 ± 0.50	3.75 ± 0.41
C	2.16	5.57	3.00	2.03	5.86	3.47	2.36	5.86	3.20	2.84	5.12	3.49	2.41 ± 0.41	5.61 ± 0.42	3.38 ± 0.16
D	3.16	3.57	5.50	4.14	3.08	4.43	3.97	3.42	4.28	3.79	2.66	5.14	3.96 ± 0.17	3.05 ± 0.38	4.62 ± 0.46
E	0.56	1.67	0.05	0.45	1.75	0.14	0.46	1.74	0.13	0.45	1.74	0.13	0.45 ± 0.00	1.74 ± 0.00	0.13 ± 0.01
F	0.36	1.87	0.40	0.32	1.87	0.34	0.32	1.87	0.35	0.36	1.83	0.28	0.34 ± 0.02	1.86 ± 0.02	0.33 ± 0.04
G	0.46	1.77	0.20	0.44	1.76	0.16	0.44	1.76	0.16	0.42	1.77	0.18	0.43 ± 0.01	1.76 ± 0.01	0.17 ± 0.01
H	0.36	1.97	0.50	0.29	1.91	0.41	0.29	1.90	0.40	0.30	1.89	0.39	0.29 ± 0.01	1.90 ± 0.01	0.40 ± 0.01

values are $\times 10^{-6}$ mol L^{-1}

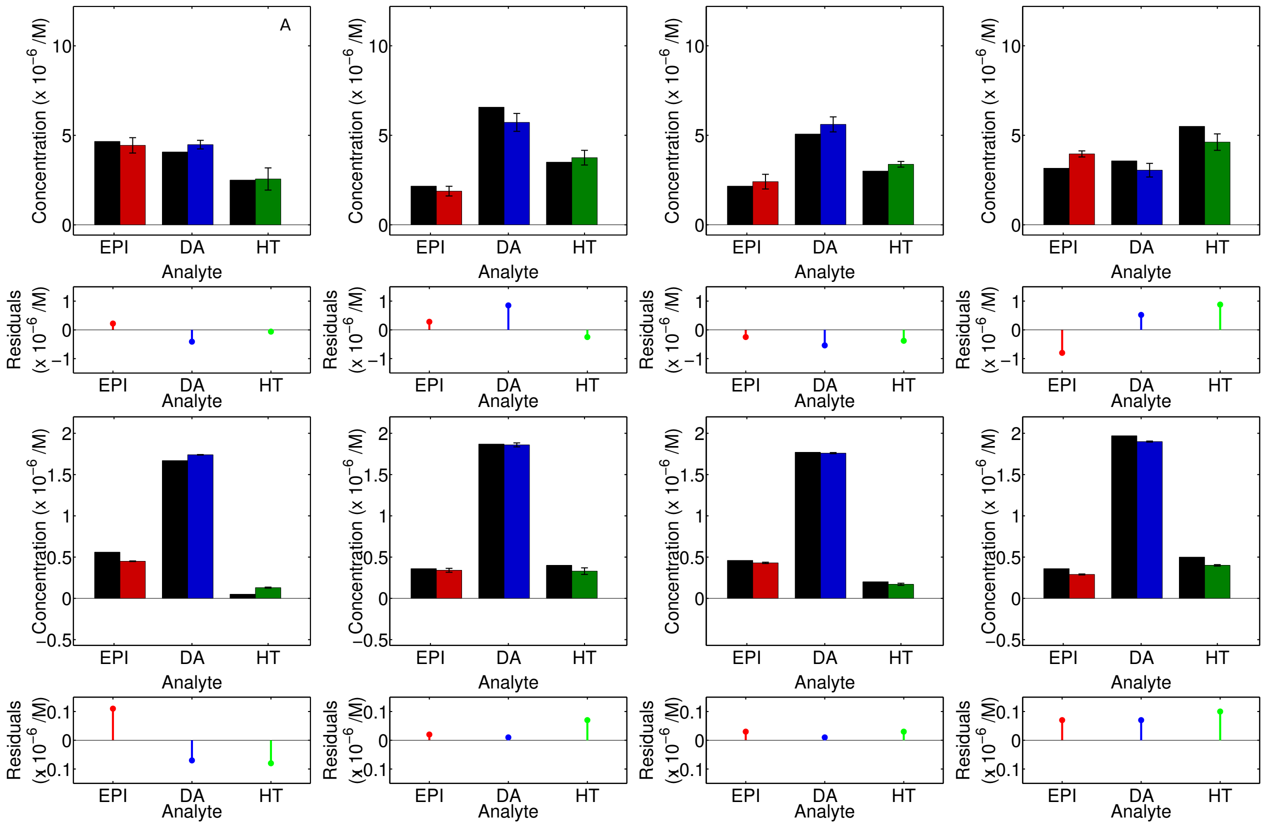

Figure S21: Independent validation of the calibration dataset with test samples (urine).

References

[1] Haynes, W.M. *CRC Handbook of Chemistry and Physics* [Haynes, W.M. (ed.)] [5-100–5-101] (CRC Press, Boca Raton, 2011)

[2] T. Baba, T. Matsui,K. Kamiya, M. Nakano and Y.Shigeta *Int. J. Quant. Chem.* (2014), DOI: 10.1002/qua.24631

[3] M.W.Schmidt, K.K.Baldridge, J.A.Boatz, S.T.Elbert, M.S.Gordon, J.H.Jensen, S.Koseki, N.Matsunaga, K.A.Nguyen, S.J.Su, T.L.Windus, M.Dupuis, J.A.Montgomery *J. Comput. Chem.* **14**, 1347-1363 (1993)

[4] F. H. Allen, *Acta Cryst.*, **B58**, 380-388 (2002)

[5] Bode, B. M. and Gordon, M. S. J. Mol. *Graphics and Modeling* **16** 133-138 (1999)

[6] Bischop M. C., *Neural Networks for Pattern Recognition* (Clarendon Press, Oxford, 1995)

[7] Wold, S., Esbensen, K., Geladi, P. Principal component analysis. *Chemom. Intell. Lab. Syst.* **2**, 37–52 (1987).

[8] De Jong, S. SIMPLS: An alternative approach to partial least squares regression. *Chemom. Intell. Lab. Syst.* **18**, 251–263 (1993).

[9] Wold, S., Sjöström, M., Eriksson, L. PLS-regression: a basic tool of chemometrics. *Chemom. Intell. Lab.* **58**, 109–130 (2001).

[10] Golbraikh, A. and Tropsha, A. Beware of q2! *J. Mol. Graphics Modell.* **20**, 269–76 (2002).
